# Supplementary material for: miR-140 Attenuates the Progression of Early-Stage Osteoarthritis by Retarding Chondrocyte Senescence
Source: Mol Ther Nucleic Acids. 2019 Nov 9;19:15–30. doi: 10.1016/j.omtn.2019.10.032 (PMC6909049; doi:10.1016/j.omtn.2019.10.032)
Supplement: Document S1. Figures S1–S6 and Tables S1–S5 [file mmc1.pdf]

OMTN, Volume *19*

Supplemental Information

miR-140 Attenuates the Progression of Early-Stage Osteoarthritis by Retarding Chondrocyte Senescence

Hai-bo Si, Ti-min Yang, Lan Li, Mei Tian, Li Zhou, Dai-ping Li, Qiang Huang, Peng-de Kang, Jing Yang, Zong-ke Zhou, Jing-qiu Cheng, and Bin Shen

SUPPLEMENTARY DATA

Table S1 General characteristics of the cartilage donors

Groups	N	Age (years)	Gender (male : female)	Side (left : right)	KL grade
Normal	5	48.0 (39, 42, 48, 53, 58)	3: 2	3 : 2	0
E-OA	5	57.8 (51, 52, 60, 61, 65)	2 : 3	3 : 2	1.8 (1, 2, 2, 2, 2)
ML-OA	5	58.6 (51, 58, 59, 62, 63)	2 : 3	2 : 3	3.6 (3, 3, 4, 4, 4)

Abbreviations: E-OA, early-stage OA; ML-OA, middle- to late-stage OA; KL, Kellgren and Lawrence X-ray criteria.

Table S2 The specific primers used for different genes

Genes		Primer
p16^{INK4a}	Forward	GGCCGATCCAGGTCATGATGATG
	Reverse	CACCAGCGTGTCCAGGAAGC
p21	Forward	GATGGAACTTCGACTTTGTCAC
	Reverse	GTCCACATGGTCTTCCTCTG
p53	Forward	TTCCTGAAAACAACGTTCTGTC
	Reverse	AACCATTGTTCAATATCGTCCG
COL2A1	Forward	GATAACAGTCTTGCCCCACTTA
	Reverse	CAAGAACAGCATTGCCTATCTG
Aggrecan	Forward	GATCCTTACCGTAAAGCCCATC
	Reverse	CTCCAGTCTCATTCTCAACCTC

MMP13	Forward	CACTTTATGCTTCCTGATGACG
	Reverse	TCTGGCGTTTTTGGATGTTTAG
ADAMTS5	Forward	GACCGATGGCACTGAATGTAGGC
	Reverse	TCTCCTCCACATACTCCGCACTTG
β-actin	Forward	5’-GAAGATCAAGATCATTGCTCCT-3’
	Reverse	5’-TGGAAGGTGGACAGTGAG-3’

Table S3 The mature sequences of miR-140-5p in various species

Gene ID	Mature names	Sequence
MIMAT0000431	hsa-mir-150-5p	CAGUGGUUUUACCCUAUGGUAG
MIMAT0000573	rno-mir-150-5p	CAGUGGUUUUACCCUAUGGUAG
MIMAT0000151	mmu-mir-150-5p	CAGUGGUUUUACCCUAUGGUAG
MIMAT0001159	gga-mir-150-5p	AGUGGUUUUACCCUAUGGUAG
MIMAT0001836	dre-mir-150-5p	CAGUGGUUUUACCCUAUGGUAG
MIMAT0002143	ssc-mir-150-5p	AGUGGUUUUACCCUAUGGUAG
MIMAT0006197	mml-mir-150-5p	CAGUGGUUUUACCCUAUGGUAG
MIMAT0006812	oan-mir-150-5p	CAGUGGUUUUACCCUAUGGU
MIMAT0012745	mdo-mir-150-5p	CAGUGGUUUUACCCUAUGGUAG
MIMAT0012926	eca-mir-150-5p	CAGUGGUUUUACCCUAUGGUAG
MIMAT0014557	tgu-mir-150-5p	CAGUGGUUUUACCCUAUGGUAG
MIMAT0015763	ppy-mir-150-5p	CAGUGGUUUUACCCUAUGGUAG
MIMAT0021765	aca-mir-150-5p	CAGUGGUUUUACCCUAUGGU

MIMAT0022552	ola-mir-150-5p	CAGUGGUUUUACCCUAUGGUAG
MIMAT0023767	cgr-mir-150-5p	CAGUGGUUUUACCCUAUGGUAG
MIMAT0025434	pol-mir-150-5p	CAGUGGUUUUACCCUAUGGUAG
MIMAT0026220	ccr-mir-150-5p	CAGUGGUUUUACCCUAUGGUAG
MIMAT0032359	ssa-mir-150-5p	CAGUGGUUUUACCCUAUGGUAG
MIMAT0035960	chi-mir-150-5p	CAGUGGUUUUACCCUAUGGUAG
MIMAT0036560	tch-mir-150-5p	CAGUGGUUUUACCCUAUGGUA
MIMAT0036719	oha-mir-150-5p	CAGUGGUUUUACCCUAUGGUAG
MIMAT0037764	cpi-mir-150-5p	CAGUGGUUUUACCCUAUGGUAG
MIMAT0038160	ami-mir-150-5p	CAGUGGUUUUACCCUAUGGUAG
MIMAT0038527	cli-mir-150-5p	CAGUGGUUUUACCCUAUGGUAG
MIMAT0039778	pal-mir-150-5p	CAGUGGUUUUACCCUAUGGUAG
MIMAT0044299	gmo-mir-150-5p	CAGUGGUUUUACCCUAUGGUA
MIMAT0046452	xla-mir-150-5p	CCAGUGGUUUUACCCUAUGGUAGG
MIMAT0047004	cpo-mir-150-5p	CAGUGGUUUUACCCUAUGGUAG
MIMAT0047653	dno--mir-150-5p	CAGUGGUUUUACCCUAUGGUAG
MIMAT0048241	ocu-mir-150-5p	CAGUGGUUUUACCCUAUGGUAG

Table S4 The target genes of hsa-miR-140-5p predicted by at least three database*

AARS	CELF2	FBXO45	KLF9	NDST1	PRRG1	SLC41A2	TSC22D2
ABCA1	CEP63	FCHO2	KLK10	NFAT5	PSRC1	SLC44A2	TSPAN12
AC011897.1	CERCAM	FGF9	LAMC1	NFE2L2	PTCD3	SLC6A6	TSSK2

AC026703.1	CMTM6	FNDC5	LAMP2	NFYA	R3HDM1	SMURF1	TTK
ACSL6	CORO2A	FOXP2	LCOR	NLK	RAB10	SNX12	TTYH2
ADAM10	CREB3L1	FXR1	LHFPL2	NPL	RABIF	SNX2	TTYH3
ADAM9	CTCF	GALC	LHX2	NPR3	RALA	SNX27	TULP4
ADAMTS5	CUL3	GALNT16	LIN9	NR1D1	RALGPS1	SOX4	UBE2I
ADCY6	DAG1	GIT1	LMNB1	NRIP1	RALGPS2	SPATA6	UBE2V1
ANK2	DCUN1D3	GLI3	LPHN2	NUCKS1	RBM39	SPOCK1	UBR5
ANKFY1	DDHD2	GNG12	LRAT	NUMBL	REST	SPRED1	VEZF1
ANKIB1	DGKI	GPR161	LRP4	OGT	RFFL	SPRY4	VTI1B
ANKRD12	DNM3	GPR85	LYSMD3	OSBPL6	RFX7	SRCAP	WASF1
ARHGAP19	DOK4	GYS1	MED13	OSTM1	RNF170	ST5	WBP1L
ARL15	DPP10	HAND2	MGAT1	OTX1	RNF19A	STC2	WDFY3
ATG14	DPYSL2	HDAC4	MICAL3	PALM2-AKAP2	ROR1	STK17A	WEE1
BACH1	DTNA	HDAC7	MIER3	PAX9	SATB2	STRADB	WNT1
BAG2	DYNC1LI2	HECTD2	MIPOL1	PDGFRA	SBNO1	SULF1	WNT9A
BAZ2B	EAF1	HEY1	MMD	PFN2	SCARB2	TBC1D12	YES1
BCL11A	EFNA4	HNRNPH3	MOB3A	PGP	SCRN1	TEAD1	YOD1
BCL2L1	EGR2	HRCT1	MSMP	PITX2	SEL1L	TFPI	ZBTB10
BCL2L2	EIF2AK2	HSPA13	MTSS1	PKN2	SEPT2	TGFBR1	ZHX1
BCL9	EIF4G2	HSPA4L	MYCBP2	PLEKHG3	SERPINB1	TIMM23	ZNF608
BIRC6	ELAVL2	IGF1R	MYO10	POLR3G	SH3GL2	TJP1	ZNF800
BMP2	EPB41L2	IGFBP5	MYO6	PPARA	SHROOM3	TLR4	ZNF827

C12orf74	ERBB2IP	IPO7	NAA20	PPP1CC	SIAH1	TMEM123
CALCOCO2	ERC2	JAG1	NAIP	PPP1R12A	SLC25A27	TMEM189-UBE2V1
CALU	FADS1	KAT2B	NAV3	PPP1R9A	SLC2A1	TMEM260
CAND1	FAM175B	KATNBL1	NCKAP1	PPTC7	SLC30A5	TNN
CAPN1	FAM214A	KBTBD2	NCOA1	PRDM1	SLC33A1	TPGS2
CCNYL1	FBN1	KLF6	NCSTN	PRR14L	SLC38A2	TRMT61B

*, The databases used in this study were miRDB, miRmap, PicTar, TargetScan, and DIANA microT-CDS.

Table S5 Kyoto Encyclopedia of Genes and Genome (KEGG) pathway analysis for predicted target genes of hsa-miR-140-5p (P < 0.05)

ID	Term	No.	P	Genes
hsa04520	Adherens junction	6	0.003	IGF1R, TJP1, TGFBR1, NLK, WASF1, YES1
hsa05200	Pathways in cancer	14	0.004	IGF1R, WNT1, BMP2, FGF9, TGFBR1, ADCY6, SLC2A1, PDGFRA, RALA, BCL2L1, WNT9A, LAMC1, GNG12, GLI3
hsa05132	Salmonella infection	6	0.007	PFN2, TJP1, DYNC1LI2, WASF1, PKN2, TLR4
hsa04144	Endocytosis	10	0.008	GIT1, IGF1R, DNM3, TGFBR1, PDGFRA, SNX2, SMURF1, SNX12, RAB10, SH3GL2
hsa04151	PI3K-Akt signaling pathway	12	0.010	IGF1R, FGF9, PDGFRA, GYS1, PKN2, CREB3L1, TLR4, TNN, EFNA4, BCL2L1, LAMC1, GNG12
hsa04810	Regulation of actin cytoskeleton	9	0.011	GIT1, PFN2, FGF9, WASF1, PDGFRA, PPP1R12A, GNG12, PPP1CC, NCKAP1
hsa04931	Insulin resistance	6	0.019	PPARA, SLC2A1, GYS1, CREB3L1, OGT, PPP1CC

hsa05166	HTLV-I infection	9	0.029	WNT1, KAT2B, EGR2, TGFBR1, ADCY6, SLC2A1, PDGFRA, BCL2L1, WNT9A
hsa04330	Notch signaling pathway	4	0.031	NCSTN, KAT2B, JAG1, NUMBL
hsa04141	Protein processing in endoplasmic reticulum	7	0.035	BAG2, HSPA4L, YOD1, NFE2L2, EIF2AK2, SEL1L, CAPN1
hsa05217	Basal cell carcinoma	4	0.042	WNT1, BMP2, WNT9A, GLI3
hsa04120	Ubiquitin mediated proteolysis	6	0.047	CUL3, UBR5, BIRC6, SIAH1, UBE2I, SMURF1

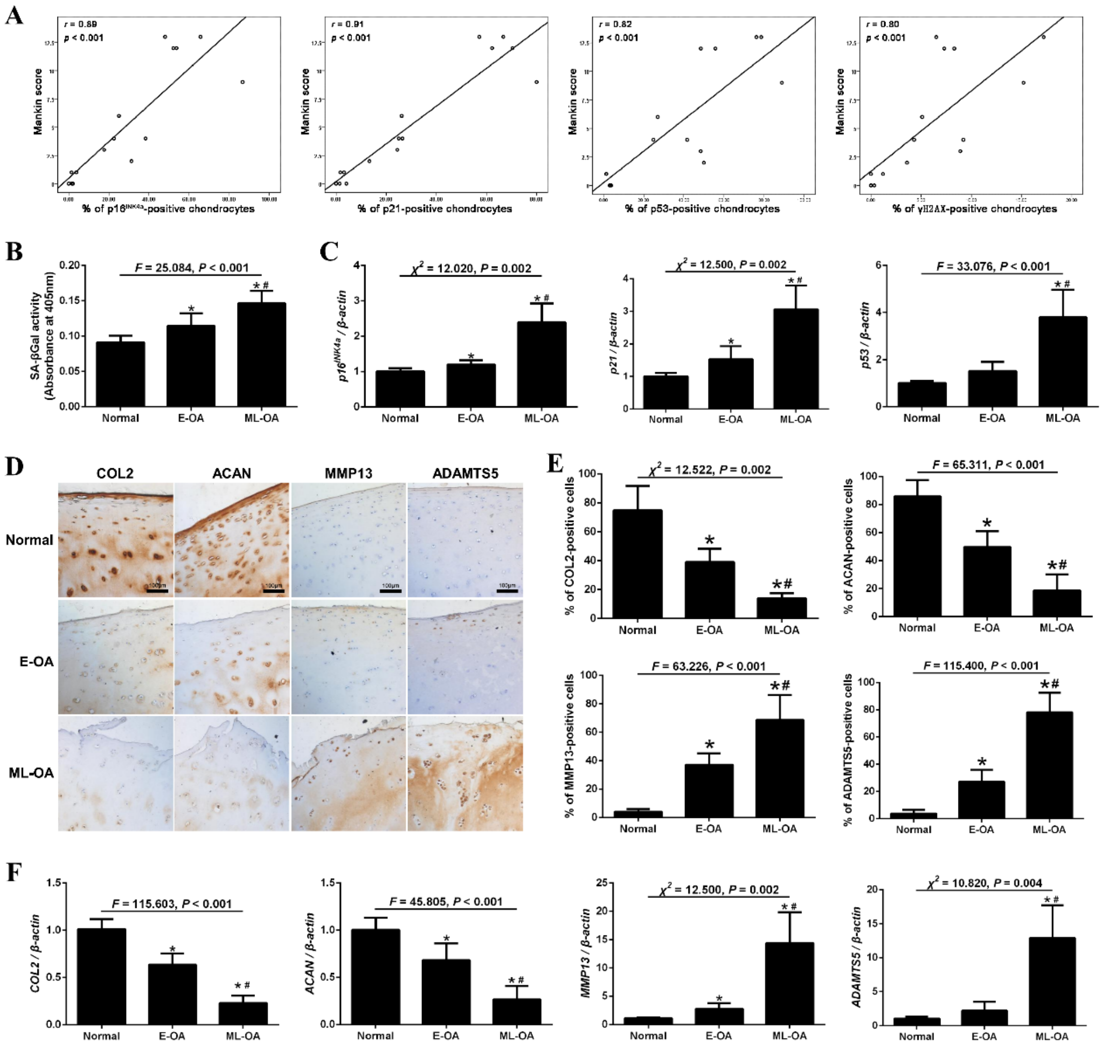

Figure S1. Chondrocyte Senescence and ECM Degradation Correlate with Human OA Pathogenesis

(A) Scatter plots of the positive correlation between the immunopositive chondrocyte percentage of p16^{INK4a}, p21, p53 and γH2AX and the modified Mankin score in normal and OA cartilage. (B-C) Basal senescence-associated β-Galactosidase (SA-βGal) activity (B) and gene expression of p16^{INK4a}, p21 and p53 (C) in normal and OA cartilage-derived chondrocytes. (D) Representative micrographs of immunohistochemical staining of COL2, ACAN, MMP13 and ADAMTS5 in normal and OA cartilage (scale bar, 100 μm). (E) Percentage of COL2-, ACAN-, MMP13- and

ADAMTS5-positive chondrocytes in normal and OA cartilage. (F) Gene expression of COL2, ACAN, MMP13 and ADAMTS5 in normal and OA cartilage-derived chondrocytes. E-OA, early-stage OA; ML-OA, middle- to late-stage OA. *$p < 0.05$ with respect to normal group, $^{\#}p < 0.05$ with respect to E-OA group.

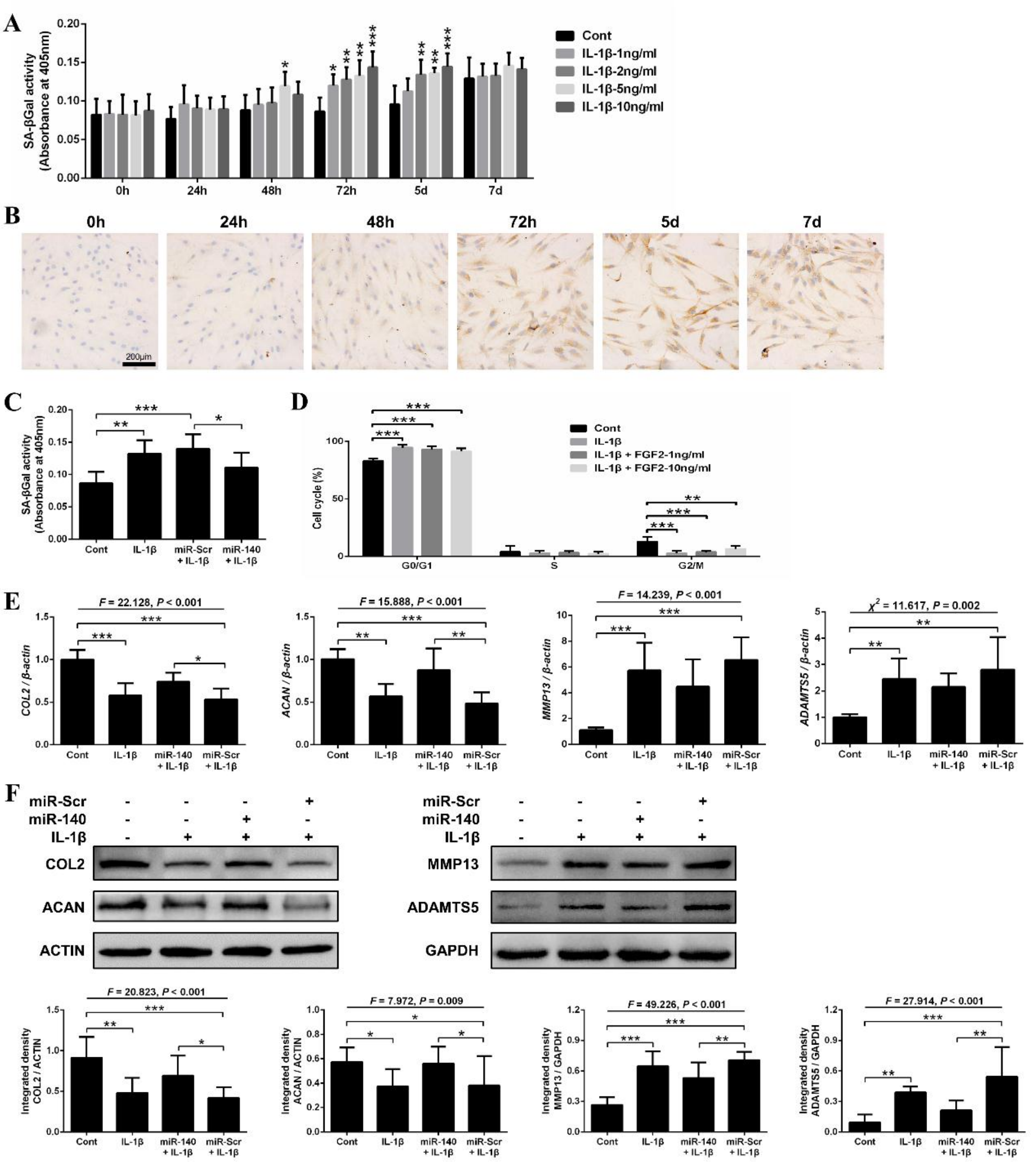

Figure S2. Pretransfection with miR-140 Inhibits IL-1β-induced Human Chondrocyte Senescence and Stabilizes ECM Homeostasis

(A-B) Optimization of IL-1β-induced *in vitro* chondrocyte senescence. *In vitro* chondrocyte senescence was induced by a 24-h IL-1β stimulation in normal chondrocytes, and the results of a time-course change in senescence-associated β-Galactosidase (SA-βGal) activity that included 0 h, 24 h, 48 h, 72 h, 5 d and 7 d after IL-1β treatment with different concentrations (1, 2, 5, 10 ng/ml) showed that 48 h after 5 ng/ml IL-1β stimulation, SA-βGal activity was significantly upregulated (A), while immunocytochemical staining showed that the expression of $p16^{INK4a}$ obviously increased 72 h after 5 ng/ml IL-1β stimulation (B). Thus, 24-h, 5 ng/ml IL-1β treatment was selected for *in vitro* chondrocyte senescence establishment, and a further 48-h incubation in fresh media was allowed before the final analysis. (C) SA-βGal activity in chondrocytes after treatment with normal media (Cont), treatment with IL-1β, pretransfection with miR-Scr + IL-1β, or pretransfection with miR-140 + IL-1β. (D) Percentage of chondrocytes in the G0/G1, S and G2/M phases of the cell cycle after treatment with normal media (Cont), IL-1β, IL-1β + FGF2-1 ng/ml, or IL-1β + FGF2-10 ng/ml. (E-F) Gene and protein expression of COL2, ACAN, MMP13 and ADAMTS5 in chondrocytes after treatment with normal media (Cont), treatment with IL-1β, pretransfection with miR-Scr + IL-1β, or pretransfection with miR-140 + IL-1β. *$p < 0.05$, **$p < 0.01$, ***$p < 0.001$.

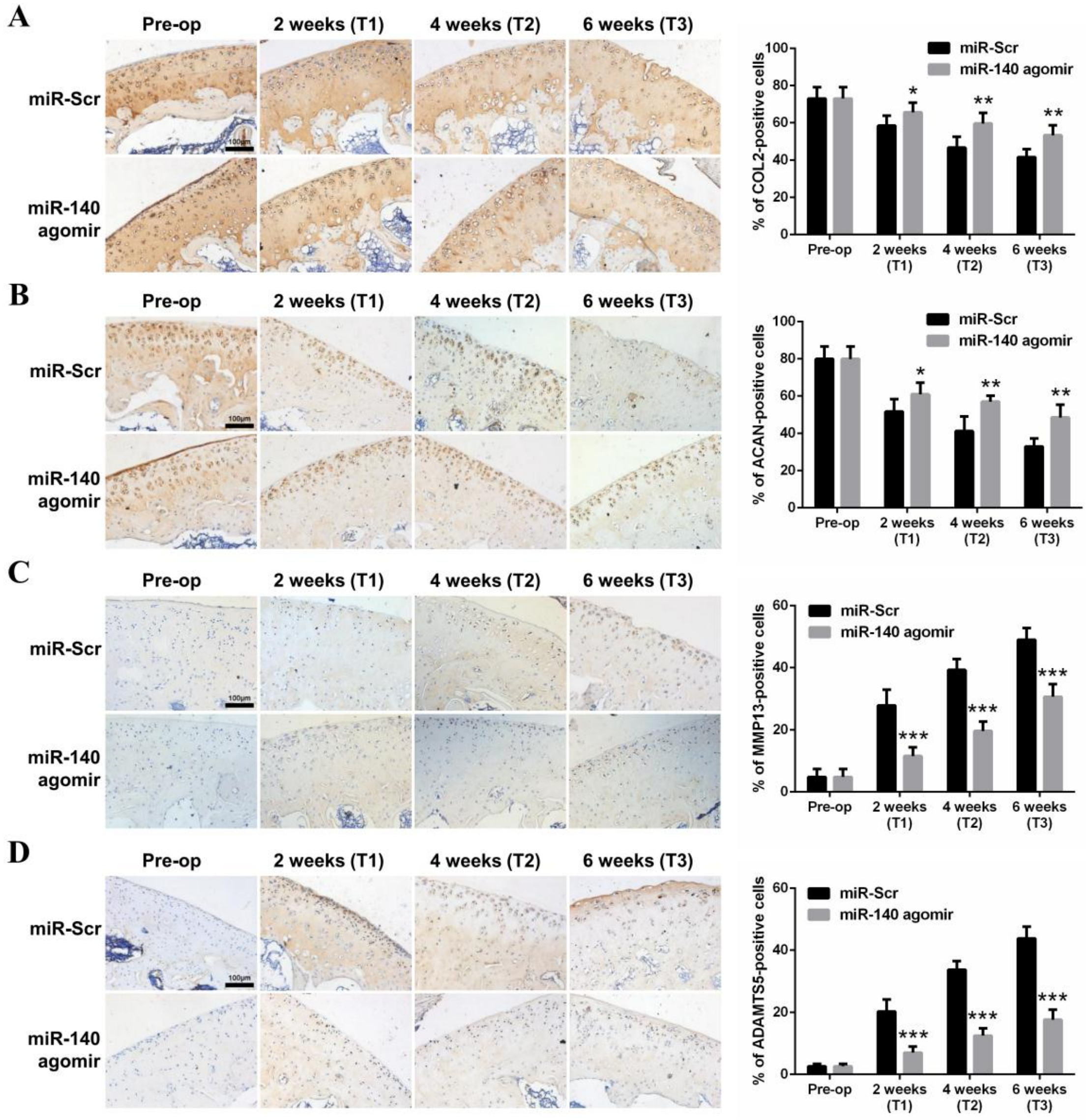

Figure S3. Intra-articular Injection (IAJ) of miR-140 Stabilizes ECM Homeostasis in Rats

(A-D) Representative micrographs of immunohistochemical staining and the immunopositive percentage of COL2 (A), ACAN (B), MMP13 (C) and ADAMTS5 (D) in sagittal sections of the medial femoral condyles after IAJ of miR-Scr or miR-140 agomir in rats (scale bar, 100 μm). * $p < 0.05$, ** $p < 0.01$, *** $p < 0.001$.

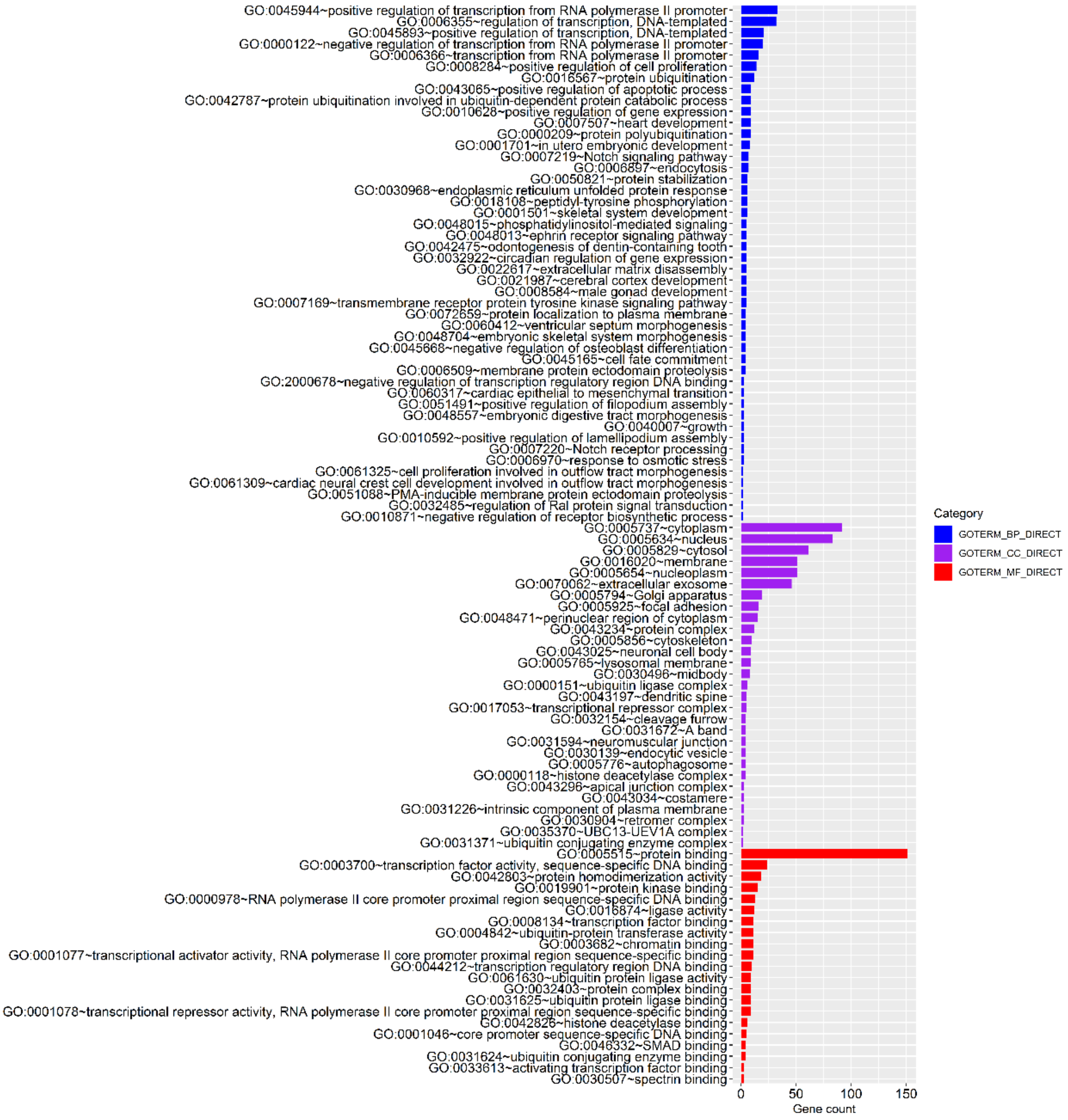

Figure S4. Gene Ontology (GO) Enrichment Analysis for the Predicted Target Genes of hsa-miR-140-5p (All Items in Each Category are Shown)

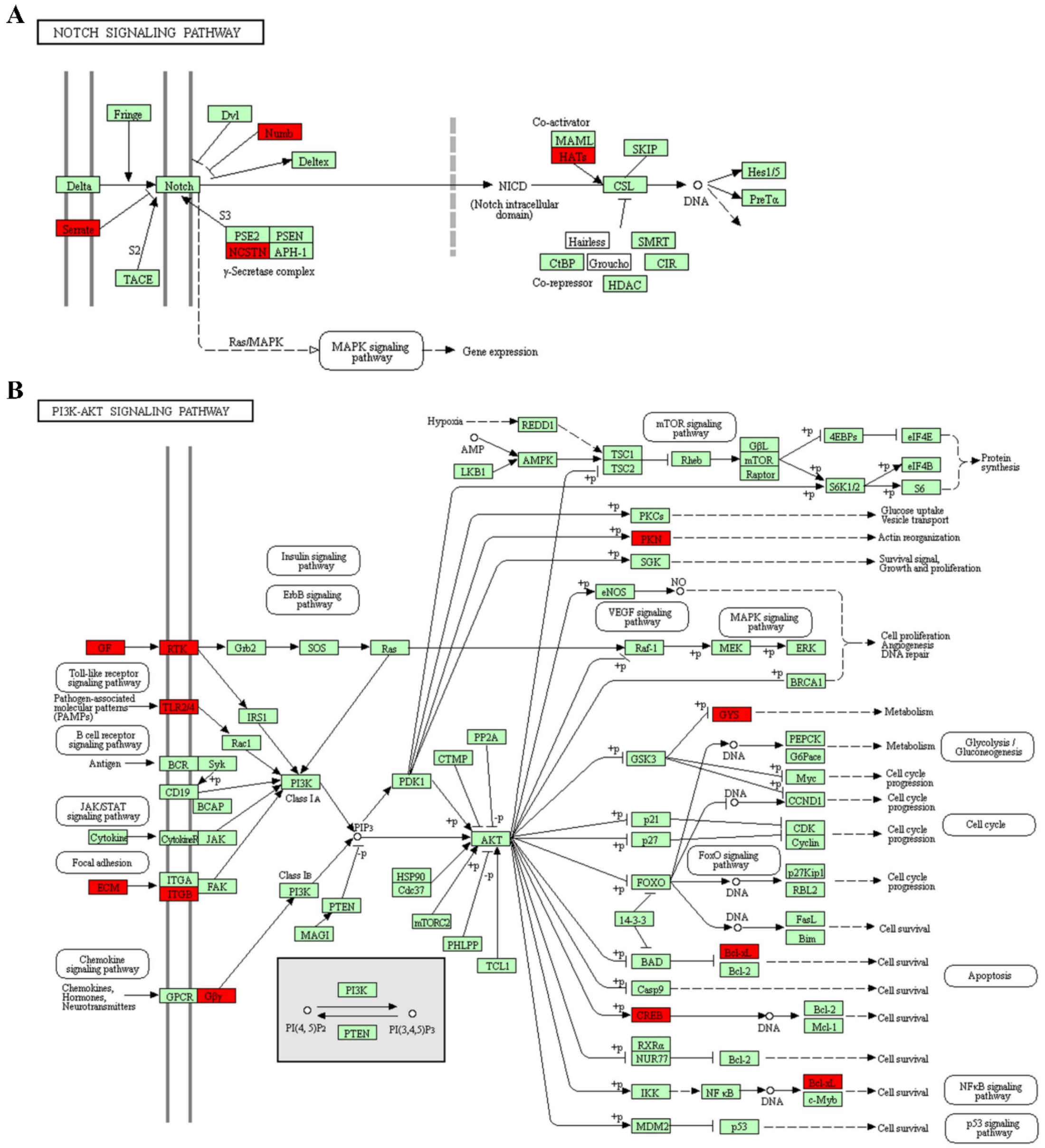

Figure S5. Signaling Pathway Information Generated by Kyoto Encyclopedia of Genes and Genomes (KEGG) Analysis

(A) Notch signaling pathway network generated by KEGG analysis. JAG1, NCSTN, KAT2B and NUMBL were predicted as target genes of hsa-miR-140-5p and enriched in the Notch pathway (located in the red region). (B) PI3K-AKT signaling pathway network generated by KEGG analysis. IGF1R, FGF9, PDGFRA, GYS1, PKN2, CREB3L1, TLR4, TNN, EFNA4, BCL2L1, LAMC1 and GNG12 were predicted as

target genes of hsa-miR-140-5p and enriched in the PI3K-AKT pathway (located in the red region).

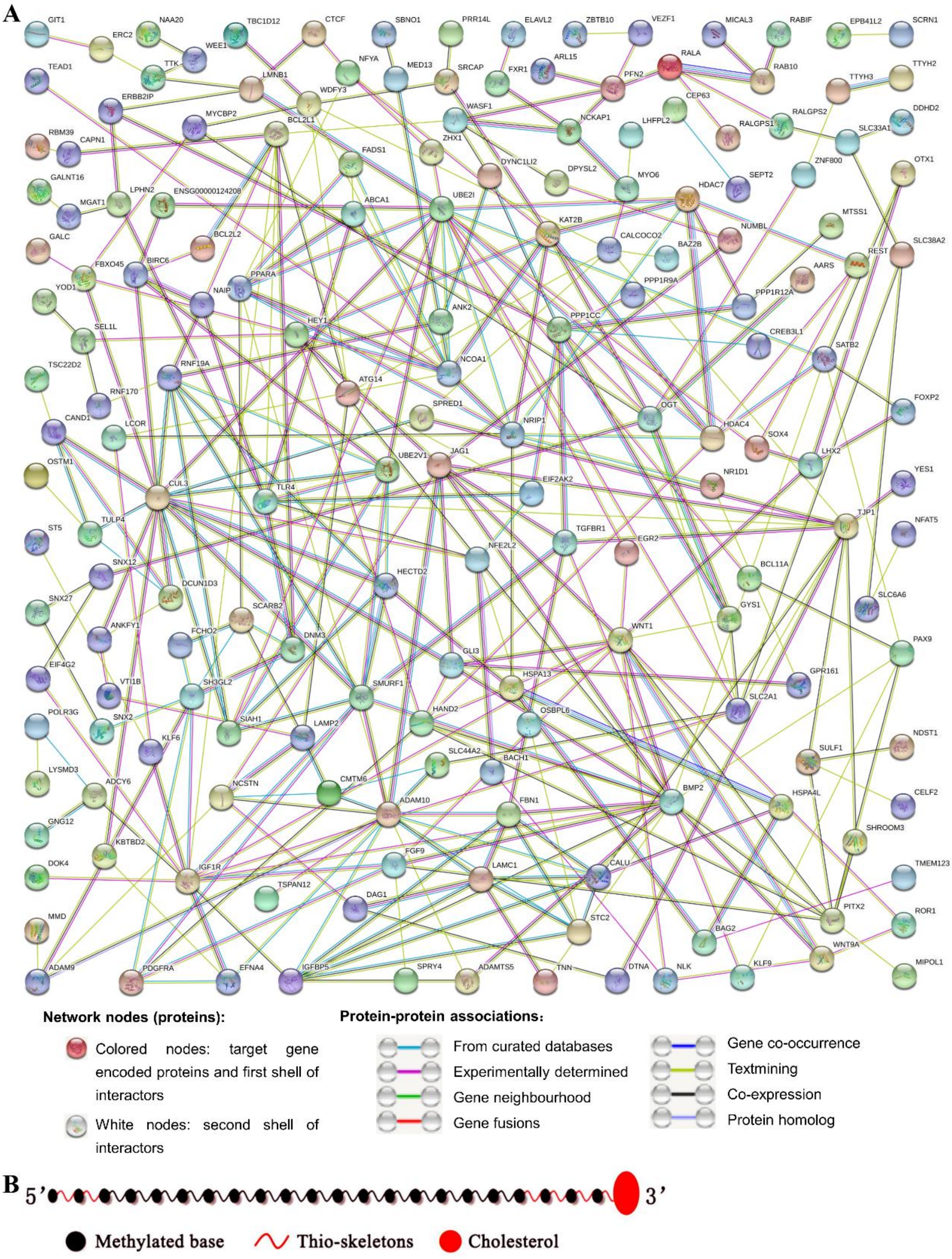

Figure S6. The Interactive Relationships among the Proteins Encoded by the

Target Genes of hsa-miR-140-5p and a Diagram of the Specifically Modified miR-140 Agomir

（A）The interactive relationships among the proteins encoded by the target genes of hsa-miR-140-5p. (B) Diagram of the specially modified hsa-miR-140 agomir. The antisense chain of the synthetic double-stranded miR-140 was fully modified by the addition of methoxyl group, the 3’ end was modified by the addition of cholesterol and thio-skeletons, and the 5’ end was modified by the addition of thio-skeletons.
