# Supplementary material for: miR-140 Attenuates the Progression of Early-Stage Osteoarthritis by Retarding Chondrocyte Senescence
Source: Mol Ther Nucleic Acids. 2019 Nov 9;19:15–30. doi: 10.1016/j.omtn.2019.10.032 (PMC6909049; doi:10.1016/j.omtn.2019.10.032)
Supplement: Document S2. Article plus Supplemental Information [file mmc2.pdf]

Molecular Therapy
Nucleic Acids
Original Article

AMERICAN SOCIETY of GENE & CELL THERAPY

miR-140 Attenuates the Progression of Early-Stage Osteoarthritis by Retarding Chondrocyte Senescence

Hai-bo Si,[1,2] Ti-min Yang,[1,3] Lan Li,[2] Mei Tian,[4] Li Zhou,[5] Dai-ping Li,[1] Qiang Huang,[1] Peng-de Kang,[1] Jing Yang,[1] Zong-ke Zhou,[1] Jing-qiu Cheng,[2] and Bin Shen[1]

[1]Department of Orthopaedics and National Clinical Research Centre for Geriatrics, West China Hospital, Sichuan University, Chengdu 610041, China; [2]Key Laboratory of Transplant Engineering and Immunology, West China Hospital, Sichuan University, Chengdu 610041, China; [3]Department of Orthopaedics, Tibet Autonomous Region People's Hospital, Lasa 850000, China; [4]Department of Ultrasound, West China Hospital, Sichuan University, Chengdu 610041, China; [5]Research Core Facility, West China Hospital, Sichuan University, Chengdu 610041, China

Osteoarthritis (OA) is a major cause of joint pain and disability, and chondrocyte senescence is a key pathological process in OA and may be a target of new therapeutics. MicroRNA-140 (miR-140) plays a protective role in OA, but little is known about its epigenetic effect on chondrocyte senescence. In this study, we first validated the features of chondrocyte senescence characterized by increased cell cycle arrest in the G0/G1 phase and the expression of senescence-associated β-galactosidase (SA-βGal), p16^{INK4a}, p21, p53, and γH2AX in human knee OA. Then, we revealed in interleukin 1β (IL-1β)-induced OA chondrocytes *in vitro* that pretransfection with miR-140 effectively inhibited the expression of SA-βGal, p16^{INK4a}, p21, p53, and γH2AX. Furthermore, *in vivo* results from trauma-induced early-stage OA rats showed that intra-articularly injected miR-140 could rapidly reach the chondrocyte cytoplasm and induce molecular changes similar to the *in vitro* results, resulting in a noticeable alleviation of OA progression. Finally, bioinformatics analysis predicted the potential targets of miR-140 and a mechanistic network by which miR-140 regulates chondrocyte senescence. Collectively, miR-140 can effectively attenuate the progression of early-stage OA by retarding chondrocyte senescence, contributing new evidence of the involvement of miR-mediated epigenetic regulation of chondrocyte senescence in OA pathogenesis.

INTRODUCTION

Osteoarthritis (OA) is a chronic and highly prevalent degenerative joint disease that mainly affects aging people and is anticipated to be the fourth leading cause of pain and physical disability by the year 2020, representing an enormous healthcare and socioeconomic burden.[1,2] However, the specific mechanisms leading to OA have not been fully elucidated, current OA treatment is mainly limited to pain management, and no effective disease-modifying therapies are available, especially in the late phase of the disease process, by which time joint arthroplasty is often indicated.[3,4] Chondrocytes are a unique cell type in articular cartilage (AC) and are solely responsible for the production and turnover of the extracellular matrix (ECM), which accounts for 95% of AC.[3,5,6] Recently, chondrocyte senescence has been suggested as an important pathological process in OA pathogenesis and may be a target of new therapeutic interventions, although the underlying mechanisms are far from being clarified.[7]

Cellular senescence refers to a signal transduction process that results in cells entering a stable state of growth arrest while remaining metabolically active.[4,8] Price et al.[9] observed senescent chondrocytes (SnCCs) near osteoarthritic lesions in the AC of OA patients but not in the AC of normal donors. Xu et al.[7] found that intra-articular injection (IAJ) of SnCCs could induce an OA-like state in the knees of mice, suggesting that chondrocyte senescence contributes to OA development and progression.[10] Moreover, SnCCs may be able to secrete various proinflammatory cytokines, catabolic enzymes, and other factors known as the senescence-associated secretory phenotype (SASP), enabling SnCCs to communicate with neighboring cells and stimulate them to senesce[8,11–13] and to interdict the synthesis of ECM components and activate proteases.[14–17] Jeon et al.[18] reported that pharmaceutical clearance of SnCCs attenuates the development of OA and creates a proregenerative environment, indicating that chondrocyte senescence is an attractive target for OA treatment. However, epigenetic strategies that can inhibit or delay chondrocyte senescence have rarely been reported.

As a result of aging and exposure to various stresses, cellular senescence is characterized by various epigenetic changes, of which the mechanisms mainly include three categories: DNA methylation, histone modifications, and regulatory microRNAs (miRNAs).[4] miRNAs are a class of single-stranded, noncoding, small RNAs, comprising 22–25 nt, and play roles in biological processes as negative regulators

Received 9 June 2018; accepted 25 October 2019;
https://doi.org/10.1016/j.omtn.2019.10.032.

Correspondence: Bin Shen, Department of Orthopaedics, West China Hospital, Sichuan University, No. 37 Guoxue Road, Chengdu 610041, China.
E-mail: shenbin_1971@163.com

This is an open access article under the CC BY license (http://creativecommons.org/licenses/by/4.0/).

of gene expression by promoting mRNA degradation and/or translational repression through sequence-specific interactions with the 3′ UTRs of specific mRNA targets.[19] One-third of all mammalian mRNA seems to be under miRNA regulation,[20,21] and increasing evidence suggests that miR-140-5p (hereafter referred to as miR-140) is mainly expressed in AC, and its level decreases in knee OA cartilage.[22,23] Although the specific mechanisms have not been elaborated, we have reported that IAJ of miR-140, at the early stage of experimental OA (E-OA), can effectively attenuate cartilage degeneration and OA progression.[19,24] Compared with the protective effect of miR-140, chondrocyte senescence plays an opposite role in OA pathogenesis, but whether miR-140 can regulate chondrocyte senescence and the potential mechanisms have never been reported.

In the current study, the features of chondrocyte senescence in normal and OA human cartilage and chondrocytes were first investigated. Then, *in vitro* and *in vivo* OA models were established, and the hypothesis that miR-140 could attenuate OA progression via protecting chondrocytes against senescence was verified. Finally, bioinformatics analysis was utilized to identify the potential mechanisms by which miR-140 regulates chondrocyte senescence. The results provide initial evidence that miR-140 can effectively attenuate OA progression by retarding chondrocyte senescence and may be a promising therapeutic agent for OA.

RESULTS

Chondrocyte Senescence Correlates with OA Pathogenesis

According to H&E and Safranin O staining of human knee cartilage (Figure 1A), the modified Mankin score was significantly higher in the OA groups than in the normal group (Figure 1B). Immunohistochemical staining showed that p16^{INK4a}, p21, and p53 were minimally expressed in normal cartilage, although remarkably increased in OA cartilage (Figure 1C). Quantitatively, the percentage of cells positive for these markers was significantly higher in the OA groups than in the normal group, and the middle- to late-stage OA (ML-OA) group showed the highest percentage of positive cells (Figures 1D–1F). p53/p21 pathways are typically thought to be activated in response to DNA damage response (DDR), and a significant increase in the expression of phospho-histone H2AX Ser139 (γH2AX) was also found in the OA groups compared with the normal group (Figures 1C and 1G). Further correlation analyses showed that the immunopositive chondrocyte percentage of the above senescence-associated markers was positively correlated with the Mankin score (Figure S1A).

Primary chondrocytes were isolated from human knee cartilage, and a significant increase in the percentage of cells in the G0/G1 phase and a significant reduction in the percentage of cells in the G2/M phase were observed in OA chondrocytes compared with normal chondrocytes (Figure 1H). Meanwhile, the percentage of senescence-associated β-galactosidase (SA-βGal)-positive cells (Figures 1I and 1J), the level of SA-βGal activity (Figure S1B), and the gene expression of p16^{INK4a}, p21, and p53 (Figure S1C) were also increased in OA chondrocytes compared with in normal chondrocytes. These results indicate that chondrocyte senescence correlates with OA pathogenesis.

Additionally, ECM homeostasis-related molecules, including the primary ECM components collagen type II (COL2) and aggrecan (ACAN) and the key matrix-degrading enzymes matrix metalloproteinase 13 (MMP13) and a disintegrin and metalloproteinase with thrombospondin motifs 5 (ADAMTS5), were also investigated. Both the immunohistochemical analysis of cartilage and the gene expression analysis of primary chondrocytes revealed significantly lower levels of COL2 and ACAN but significantly higher levels of MMP13 and ADAMTS5 in the OA groups than in the normal group (Figures S1D–S1F).

Pretransfection with miR-140 Inhibits IL-1β-Induced Chondrocyte Senescence

A 24-h, 5 ng/mL interleukin-1β (IL-1β) treatment, followed by 48 h incubation in fresh media, was selected for the induction of OA-like chondrocyte senescence (Figures S2A and S2B). Compared with nonstimulated chondrocytes (control group), IL-1β stimulation reduced the miR-140 level in chondrocytes (Figure 2A), arrested chondrocytes in the G0/G1 phase (Figure 2B), and induced increases in the percentage of SA-βGal-positive cells (Figure 2C); SA-βGal activity (Figure S2C); gene and protein expression of p16^{INK4a}, p21, and p53 (Figures 2D and 2E); and the percentage of γH2AX-positive cells (Figure 2F). The differences between the control and IL-1β groups were all statistically significant except for p53 gene expression. IL-1β-induced cell cycle arrest in the G0/G1 phase was not significantly improved, even with fibroblast growth factor 2 (FGF2) stimulation (Figure S2D), indicating that IL-1β stimulation can induce irreversible chondrocyte senescence.

Pretransfection with miR-140 increased the intracellular miR-140 level in chondrocytes and reduced the proportion of chondrocytes in the G0/G1 phase; the percentage of SA-βGal- and γH2AX-positive cells; SA-βGal activity; and the gene and protein expression of p16^{INK4a}, p21, and p53 (Figures 2 and S2C). The differences in the proportion of chondrocytes in the G0/G1 phase, SA-βGal activity, the percentage of SA-βGal- and γH2AX-positive cells, and the protein expression of p16^{INK4a} and p21 between the miR-Scr + IL-1β and miR-140 + IL-1β groups reached statistical significance. These findings indicate that IL-1β-induced chondrocyte senescence can be, at least in part, retarded by pretransfection with miR-140.

As shown in Figures S2E and S2F, the gene and protein expression of COL2 and ACAN significantly decreased, whereas MMP13 and ADAMTS5 significantly increased in IL-1β-stimulated chondrocytes compared with nonstimulated chondrocytes. Moreover, COL2 and ACAN were significantly upregulated at both the gene and protein levels, whereas MMP13 and ADAMTS5 were significantly downregulated at the protein level in the miR-140 + IL-1β group compared with the miR-Scr + IL-1β group, suggesting that pretransfection with miR-140 also protects chondrocytes against IL-1β-induced ECM degradation.

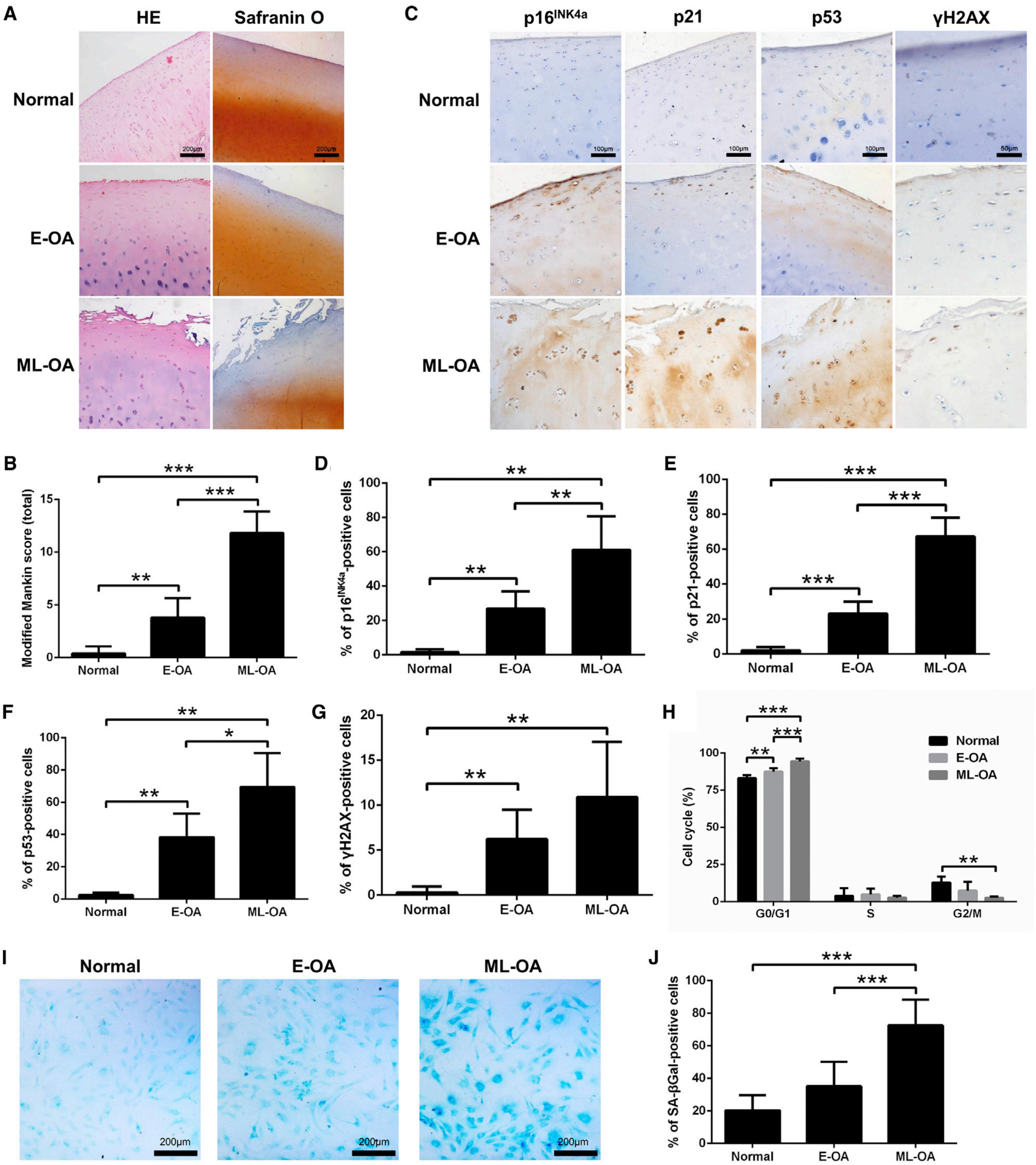

Figure 1. Chondrocyte Senescence Correlates with Human OA Pathogenesis

(A) Representative micrographs of H&E and Safranin O staining of normal and OA cartilage (scale bars, 200 μm). (B) The modified Mankin score of normal and OA cartilage. (C) Representative micrographs of immunohistochemical staining of p16^{INK4a}, p21, p53, and γH2AX in normal and OA cartilage (scale bars, 100 μm for p16^{INK4a}, p21, and p53; 50 μm for γH2AX). (D–G) The percentage of (D) p16^{INK4a}-, (E) p21-, (F) p53-, and (G) γH2AX-positive chondrocytes in normal and OA cartilage. (H) Percentage of normal and OA cartilage-derived chondrocytes in the G0/G1, S, and G2/M phases of the cell cycle, determined by flow cytometric analysis. (I) Representative micrographs of SA-βGal staining in normal and OA cartilage-derived chondrocytes (scale bars, 200 μm). (J) Percentage of normal and OA cartilage-derived chondrocytes positive for SA-βGal. E-OA, early-stage OA; ML-OA, middle- to late-stage OA. *$p < 0.05$, **$p < 0.01$, ***$p < 0.001$.

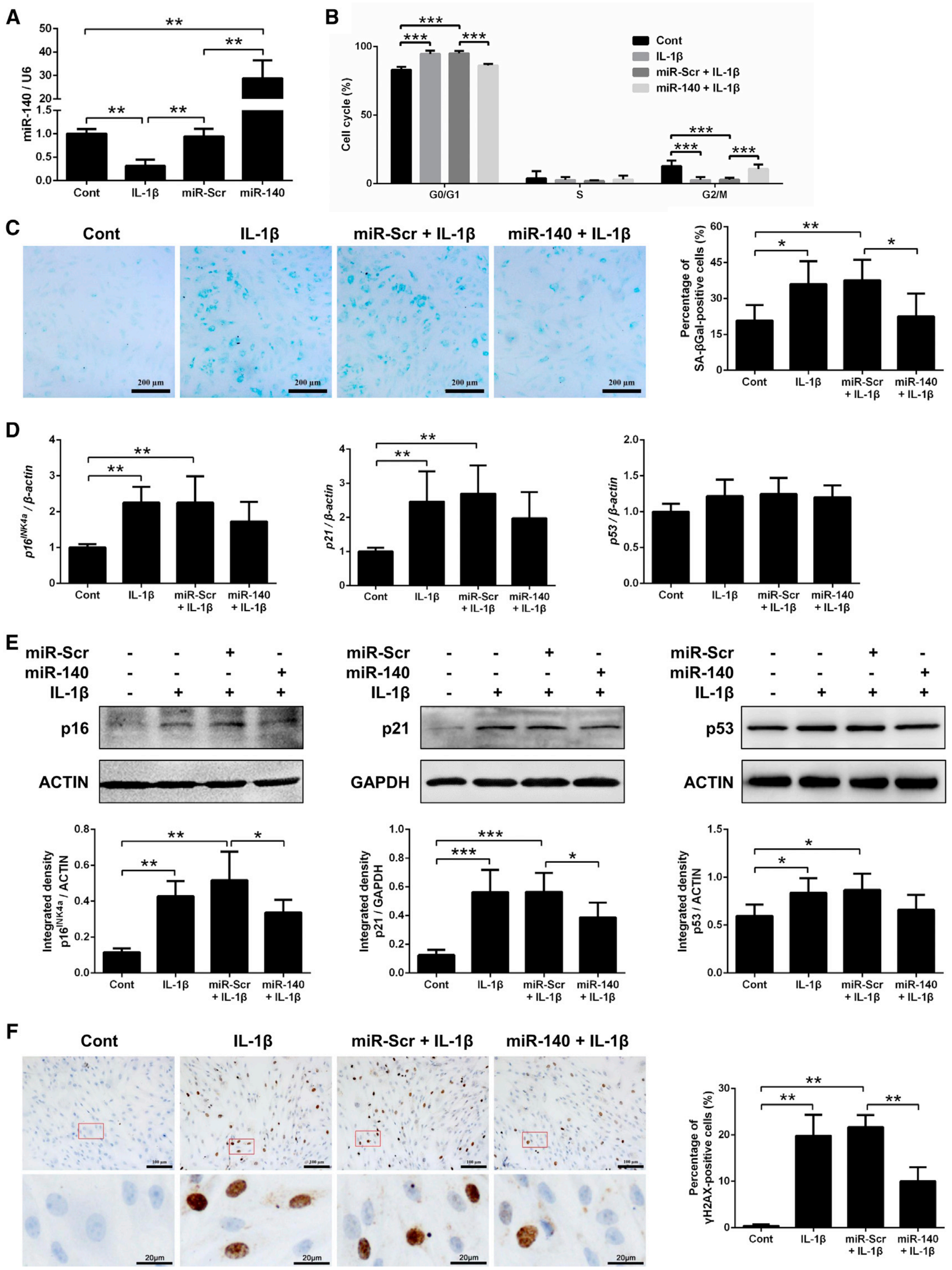

Figure 2. Pretransfection with miR-140 Inhibits IL-1β-Induced Human Chondrocyte Senescence

(A) PCR analysis of the relative expression level of miR-140, 24 h after IL-1β stimulation or transfection with miR-Scr or miR-140 in normal cartilage-derived chondrocytes. (B–F) Percentage of chondrocytes in the G0/G1, S, and G2/M phases of the cell cycle (B), representative micrographs of SA-βGal staining (scale bars, 200 μm) and percentage of SA-βGal-positive chondrocytes (C), gene (D) and protein (E) expression of $p16^{INK4a}$, p21, and p53, representative micrographs of γH2AX staining (scale bars, top: 100 μm; bottom: 20 μm), and the percentage of γH2AX-positive chondrocytes (F) after treatment with normal media (Cont), treatment with IL-1β, pretransfection with miR-Scr + IL-1β, or pretransfection with miR-140 + IL-1β. $^{*}p < 0.05$, $^{**}p < 0.01$, $^{***}p < 0.001$.

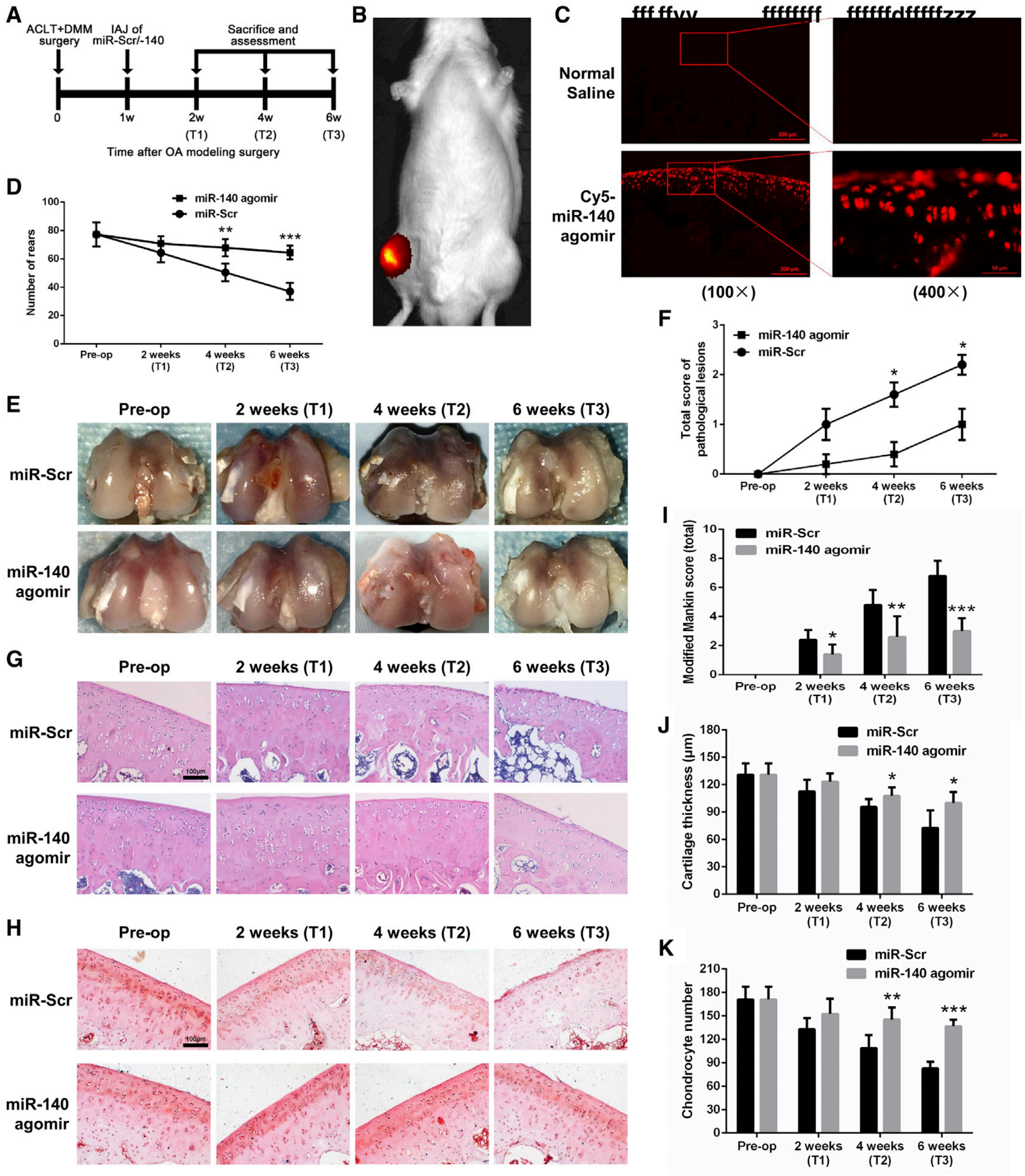

Figure 3. Intra-articular Injection (IAJ) of miR-140 Attenuates OA Progression in Rats

(A) Experimental layout for the *in vivo* experiments in rats. (B) Representative *in vivo* fluorescence image 24 h after IAJ of Cy5-labeled miR-140 agomir into the right hind knee and normal saline (NS) injection into the left hind knee. (C) Representative fluorescence micrographs of sagittal frozen sections of the medial femoral condyles 24 h after IAJ of NS into the left hind knee (top) and Cy5-labeled miR-140 agomir into the right hind knee (bottom; 100× scale bars, 200 μm; 400× scale bars, 50 μm). (D) Number of rears, as measured by behavioral testing, before and after IAJ of miR-Scr or miR-140 agomir. (E–K) Gross observation (E), pathological lesion score (F), representative sagittal micrographs of H&E (G), and Safranin O (H) staining (scale bars, 100 μm) and modified Mankin score (I), cartilage thickness (J), and chondrocyte number (K) of the medial femoral condyles before and after IAJ of miR-Scr or miR-140 agomir. ACLT + DMM, anterior cruciate ligament transection (ACLT) and destabilization of the medial meniscus (DMM). *p < 0.05, **p < 0.01, ***p < 0.001.

IAJ of miR-140 Attenuates OA Progression in Rats

The rat OA model was established by the classical anterior cruciate ligament transection (ACLT) + destabilization of the medial meniscus (DMM) method, and the effect of miR-140 on chondrocyte senescence was investigated *in vivo* (Figure 3A). As shown in Figure 3B, strong fluorescence was observed in and around the right knee region where Cy5-labeled miR-140 agomir was injected, whereas no fluorescence was detected in the left knee region where normal saline (NS)

was injected. No fluorescence was observed in other parts of the body. Furthermore, frozen sections of bilateral femoral condyles were made and used for fluorescence imaging, and strong fluorescence was observed in the cytoplasm of almost all cartilaginous chondrocytes in the right femoral condyle, whereas no fluorescence was observed in the left femoral condyle (Figure 3C), thus visually confirming that the intra-articularly injected miR-140 can rapidly enter the cartilage and reach the chondrocyte cytoplasm.

The surgical wound healed well within 1 week postoperatively, and no complications were observed throughout the observational period in any rats. As shown in Figure 3D, the number of rears, an important indicator of joint pain and spontaneous activity levels, was higher in the miR-140 group than in the miR-Scr group at T1 to T3, and the differences at T2 and T3 were statistically significant. The gross appearance of the medial femoral condyles is shown in Figure 3E. The cartilage surface was smooth, and no irregularities or pits were observed in normal rats; these rats even showed a relatively normal surface at T1. Fibrillation, surface erosion, pitting, ulceration, or osteophytes were observed at T2 and T3, and the miR-140 group displayed less extensive cartilage destruction than the miR-Scr group. Quantitatively, the pathological lesion score was lower in the miR-140 group than in the miR-Scr group, and statistically significant differences were observed at T2 and T3 (Figure 3F).

The histological changes of the cartilage were further investigated by H&E and Safranin O staining (Figures 3G and 3H), and the Mankin score was significantly lower in the miR-140 group than in the miR-Scr group at T1 to T3 (Figure 3I). The cartilage thickness and the number of chondrocytes were also higher in the miR-140 group than in the miR-Scr group at T1 to T3 (Figures 3J and 3K), and the differences at T2 and T3 were statistically significant. These results suggest that IAJ of miR-140 in the early stage of OA effectively attenuates OA progression in rats.

IAJ of miR-140 Retards Chondrocyte Senescence in Rats

To localize SnCCs in rat cartilage, immunohistochemical analyses for senescence-associated markers, as in human cartilage, were performed. As shown in Figure 4, there were little to no $p16^{INK4a}$-, p21-, p53-, and γH2AX-positive chondrocytes in normal (pre-op) cartilage, whereas the percentage of immunopositive chondrocytes progressively increased over time following surgery. Compared with the miR-Scr group, there were fewer $p16^{INK4a}$-, p21-, p53-, and γH2AX-positive chondrocytes in the miR-140 group at T1 to T3, and the differences in $p16^{INK4a}$ and p21 at T1 to T3, p53 at T2 and T3, and γH2AX at T3 reached statistical significance.

The percentages of COL2- and ACAN-positive chondrocytes progressively decreased with OA progression and were significantly higher, whereas the percentage of MMP13- and ADAMTS5-positive chondrocytes increased and were significantly lower in the miR-140 group than in the miR-Scr group at T1 to T3 (Figure S3). These results indicate that IAJ of miR-140 can effectively attenuate OA progression in rats via stabilizing ECM homeostasis in addition to delaying chondrocyte senescence.

Bioinformatics Analyses of the Potential Mechanisms by which miR-140 Regulates Chondrocyte Senescence

The gene ID of hsa-miR-140-5p is MIMAT0000431, and its mature sequence is 5′-CAGUGGUUUUACCCUAUGGUAG-3′, which is highly conserved in various species and consistent with that of rno-miR-140-5p (Table S3). As shown in Figure 5A, the number of predicted target genes of hsa-miR-140-5p in the miRDB, miRmap, PicTar, TargetScan, and DIANA microT-CDS databases was 413, 1,006, 223, 434, and 304, respectively. A total of 242 target genes were predicted by at least three databases and were used for further analyses (Table S4).

Gene Ontology (GO) enrichment analysis revealed that the target genes are mainly located in the cytoplasm, cytosol, membrane, etc. These genes participate in the molecular functions of protein binding, sequence-specific DNA binding, protein ligase activity, etc., and are involved in various biological processes, including regulation of transcription, protein polyubiquitination, signal transduction, etc. (Figures 5B and Figure S4). Enriched signaling pathways for the target genes of hsa-miR-140-5p were further identified by Kyoto Encyclopedia of Genes and Genomes (KEGG) pathway analysis and ranked according to the p values, and 12 pathways were statistically significant (Figure 5C; Table S5). Among them, the Notch and phosphatidylinositol 3-kinase (PI3K)-AKT pathways are well known to be associated with OA pathogenesis, and the related molecular networks are shown in Figure S5.

The STRING database was used to predict the functional protein association networks of the target genes; many of the proteins encoded by the predicted target genes of hsa-miR-140-5p interacted with each other (Figure S6A); and part of them, including JAG1 and NUMBL in the Notch pathway (Figure 5D) and insulin-like growth factor 1 receptor (IGF1R) and Toll-like receptor 4 (TLR4) in the PI3K-AKT pathway (Figure 5E), were experimentally validated by western blotting. Finally, brief schematic diagrams were constructed and explained the involvement of chondrocyte senescence in OA pathogenesis and the potential mechanistic network by which miR-140 regulates chondrocyte senescence (Figure 6).

DISCUSSION

OA is one of the most prevalent joint diseases affecting millions of people worldwide, but its pathogenesis has not been fully illuminated, and no effective disease-attenuating or -reversing interventions are currently in clinical use.[25] In this study, we first validated in human cartilage that chondrocyte senescence correlates with OA pathogenesis. Second, we found in an IL-1β-induced *in vitro* OA model in which pretransfection with miR-140 can partially retard chondrocyte senescence. Third, the *in vivo* results from a trauma-induced OA model showed that IAJ of miR-140 could induce molecular changes similar to those observed *in vitro*, resulting in a noticeable alleviation of OA progression. Finally, the bioinformatics analysis predicted the

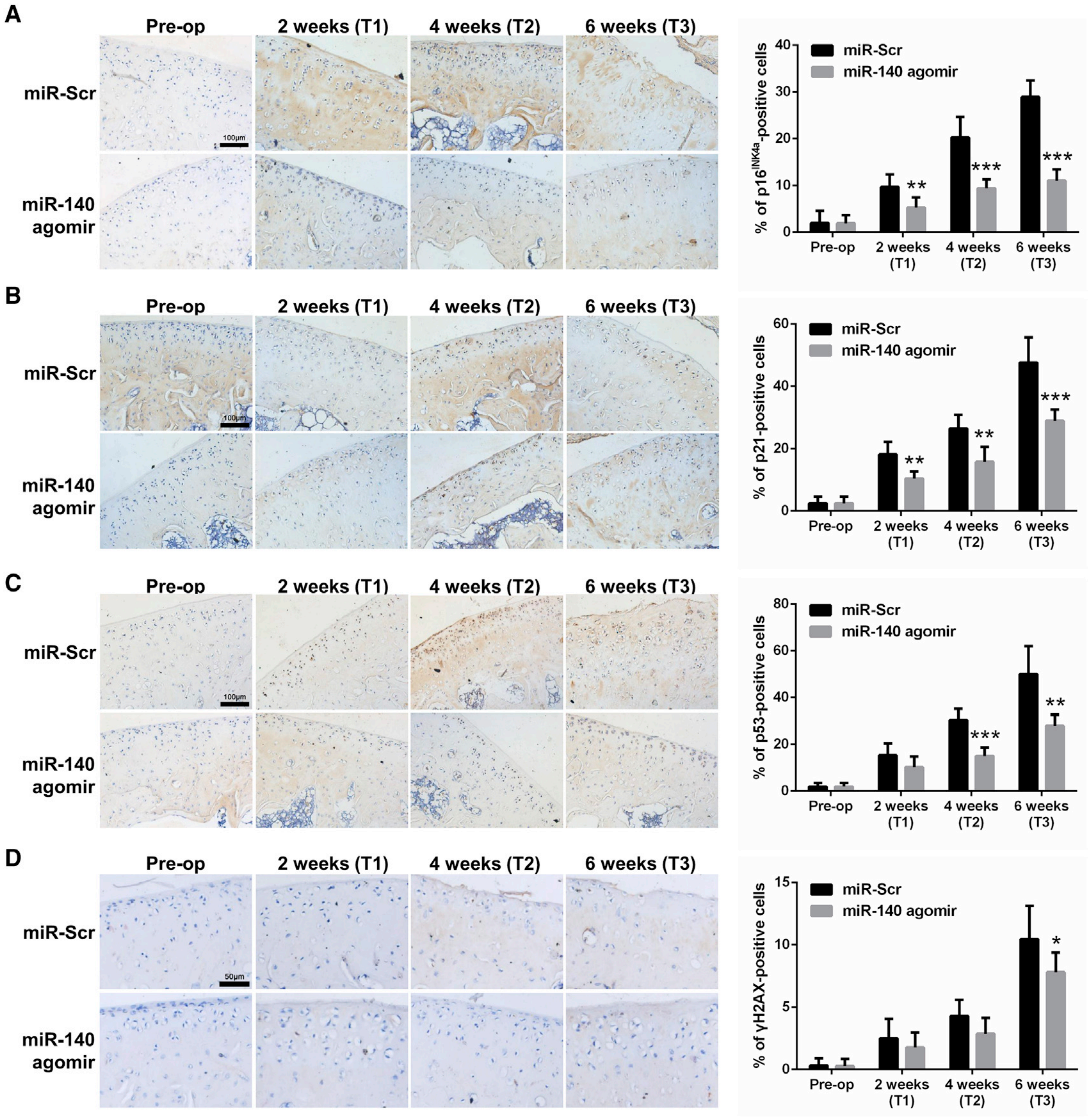

Figure 4. Intra-articular Injection (IAJ) of miR-140 Retards Chondrocyte Senescence in Rats

(A–D) Representative micrographs of immunohistochemical staining and immunopositive percentage of $p16^{INK4a}$ (A), p21 (B), p53 (C), and γH2AX (D) in sagittal sections of the medial femoral condyles after IAJ of miR-Scr or miR-140 agomir (scale bars, 100 μm for $p16^{INK4a}$, p21, and p53; 50 μm for γH2AX). $*p < 0.05$, $**p < 0.01$, $***p < 0.001$.

potential targets of miR-140, and a possible mechanistic network by which miR-140 regulates chondrocyte senescence was constructed. The findings of this study not only highlight that miR-140 can effectively attenuate the progression of E-OA by retarding chondrocyte senescence but also contribute new evidence of the involvement of miR-mediated epigenetic regulation of chondrocyte senescence in OA pathogenesis, providing novel potential targets for OA therapeutics.

SA-βGal is a classical indicator of senescence. Gao et al.[26] demonstrated that SA-βGal expression in AC is positively correlated with progressive knee OA joint damage, and our study yielded a similar result. However, SA-βGal may not be regarded as a unique identifier of SnCCs. The key pathways involved in chondrocyte senescence are the $p16^{INK4a}$/Rb and p53/p21 pathways (Figure 6). Upregulation of $p16^{INK4a}$, in response to various stresses, leads to inhibition of cyclin-dependent kinases (CDKs) that prevent retinoblastoma protein (pRB) phosphorylation and inactivation, causing cell cycle arrest and senescence, whereas activation of p53 induces either growth arrest or apoptosis, in part, by inducing the expression of p21, which is another CDK inhibitor. Moreover, $p16^{INK4a}$ plays an important role in cell cycle regulation via decelerating progression from the G1 phase to the S phase,[27,28] and p21 is a major target of p53 and is thus associated with linking DNA damage to cell cycle arrest.[29] Therefore, $p16^{INK4a}$ has been considered more involved in the maintenance of the senescent phenotype, whereas p21 is critical for

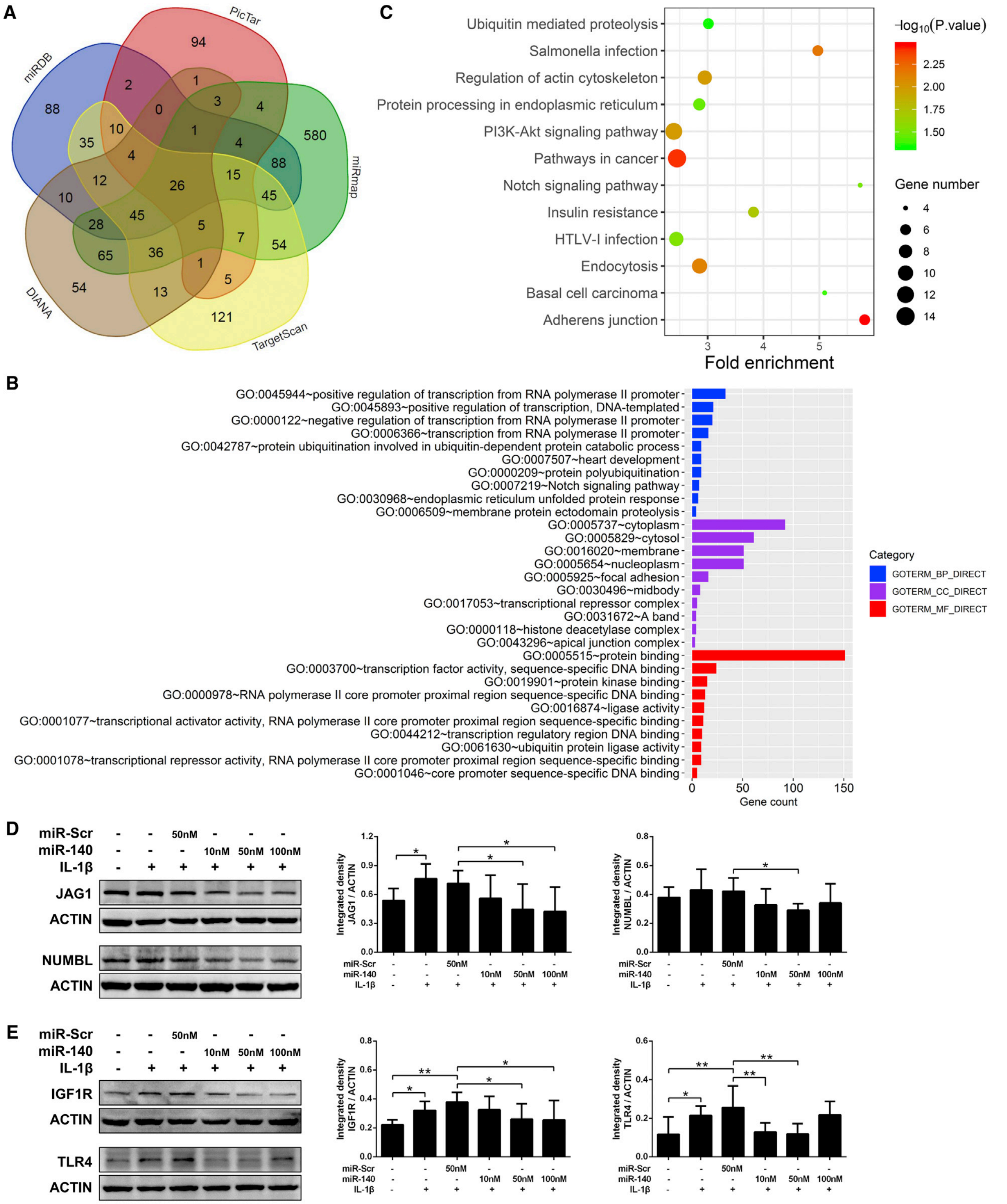

(legend on next page)

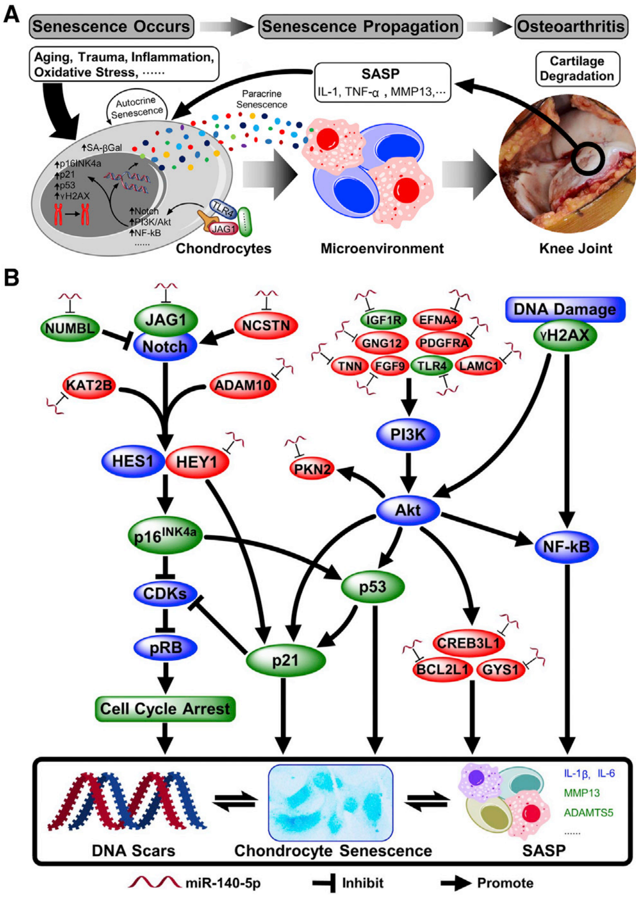

Figure 6. Schematic Diagrams of Chondrocyte Senescence and miR-140-Related Mechanisms in OA Pathogenesis

(A) Involvement of chondrocyte senescence in OA pathogenesis. (B) Potential mechanistic network (mainly Notch and PI3K-AKT pathways, which are well known to be associated with OA pathogenesis) by which miR-140 regulates chondrocyte senescence. The red nodes represent the bioinformatically predicted targets of miR-140, and the green nodes represent the validated targets of miR-140 and molecules that can be regulated by miR-140 in this study.

establishing senescence.[27–29] In this study, positive correlations of the levels of p16^{INK4a}, p21, and p53 and the DNA damage marker γH2AX with OA severity were identified, strengthening the evidence that chondrocyte senescence correlates with OA pathogenesis.

Intrinsic telomere-dependent replicative senescence and extrinsic stress-induced senescence are two main types of senescence, and failure of repair responses, due to chondrocyte senescence, would result in progressive cartilage degeneration.[10,30] As chondrocytes in the AC of adults do not normally proliferate,[31] it is unlikely that chondrocyte senescence results from multiple cycles of cell proliferation; therefore, repetitive stress stimulation may be the main cause.[10,32] In addition to the spontaneous senescence of aging, many stressful events, such as inflammation, oxidative stress, and trauma, have been implicated in extrinsic stress-induced senescence.[33] In particular, proinflammatory cytokines, such as IL-1β and trauma, can contribute to an imbalance between anabolic and catabolic mechanisms, which may also result in extrinsic stress-induced senescence.[12,13,22,34] We also verified in this study that chondrocyte senescence and an imbalance in ECM homeostasis were obviously induced by IL-1β stimulation *in vitro* and by joint trauma *in vivo*. Furthermore, the SASP is considered to be the main mechanism by which persistent, prolonged senescence occurs, even after the initial stimulus has been removed.[3,4,33] Therefore, early blockage or inhibition of chondrocyte senescence is a key consideration in developing new therapeutic protocols for OA.

Jeon et al.[18] reported that selective elimination of SnCCs attenuated the development of post-traumatic OA, reduced pain, and increased cartilage development. These results were further validated in transgenic, nontransgenic, and aged mice via IAJ of a senolytic compound (UBX0101) or a molecule that induces apoptosis in p16^{INK4a}-expressing cells (AP20187) that selectively killed SnCCs,[18] indicating that chondrocyte senescence is an attractive target for OA treatment. In this study, we mainly focused on epigenetic strategies that can inhibit or delay chondrocyte senescence, which may be a promising approach for treating OA, because unlike genetic changes, epigenetic changes can be reversed and are easily controlled.[24,35] miR-140 plays a critical epigenetic role in OA pathogenesis,[19] and previous studies have confirmed that the miR-140 level is decreased in human knee OA cartilage and is negatively correlated with OA severity,[22,23,36] which seems to oppose the trends of other senescence-related markers, suggesting that epigenetic mechanisms between miR-140 and chondrocyte senescence may exist. As expected, we found that IL-1β- and trauma-induced chondrocyte senescence could be inhibited, at least in part, by pretreatment with miR-140, which has rarely been reported. We also discovered that the potential target genes of miR-140 were enriched in various biological processes, such as metabolic processes, cell proliferation, signal transduction, and response to stress, and were mainly located within 10 pathways, among which the Notch and PI3K-AKT signaling pathways are well known to be involved in OA pathogenesis.

Notch is a single-pass transmembrane cell surface receptor that plays a critical role in cell fate via regulating differentiation and apoptosis.[37]

Figure 5. Bioinformatics Analyses of the Potential Mechanisms by which miR-140 Regulates Chondrocyte Senescence

(A) The number of predicted target genes of hsa-miR-140-5p in the miRDB, miRmap, PicTar, TargetScan, and DIANA microT-CDS databases. (B) Gene Ontology (GO) enrichment analysis for the predicted target genes of hsa-miR-140-5p, showing only the top 10 items in each category; the detailed results are provided in Figure S4. (C) Kyoto Encyclopedia of Genes and Genomes (KEGG) pathway analysis for the predicted target genes of hsa-miR-140-5p ($p < 0.05$). (D and E) Experimental validation of the effect of miR-140 on the proteins encoded by the predicted target genes of hsa-miR-140-5p by western blotting, including JAG1 and NUMBL in the Notch pathway (D) and IGF1R and TLR4 in the PI3K-AKT pathway (E). *$p < 0.05$, **$p < 0.01$.

Recent data point to a role for the Notch pathway in the dynamic control of both SASP composition and its net functional output;[38] for example, pharmacological inhibition of Notch signaling is able to reduce senescence features in esophageal keratinocytes.[39] JAG1 is a canonical Notch ligand that is remarkably increased during OA development,[40] and the JAG1-mediated Notch pathway is initiated when JAG1 binds to receptors. Upon ligand binding, the receptor is cleaved by ADAM and subsequently by a γ-secretase complex. Then, the Notch-intracellular domain translocates to the nucleus and forms a transcriptional activator that induces the HEY/HES family. Downstream signaling molecules, such as HEY1 and HES1, mediate the catabolic effect of Notch signaling in chondrocytes, inducing the expression of many inflammatory cytokines and proteolytic enzymes, such as IL-1 receptor-like 1 (IL1RL1), MMP13, and ADAMTS5.[40,41] A previous bioinformatics analysis revealed that the 3′ UTRs of the key molecules in the Notch pathway, such as JAG1, ADAM10, and HEY1, possess miR-140 binding sites.[42] In our study, JAG1, NCSTN, KAT2B, and NUMBL were predicted as targets of miR-140 and were enriched in the Notch pathway, whereas ADAM10 and HEY1 were also predicted as targets of miR-140, although they were not enriched in the Notch pathway, according to the KEGG pathway analysis. This finding implies that one of the possible mechanisms by which miR-140 retards chondrocyte senescence is the regulation of the functions of the Notch pathway (Figure 6).

Another major pathway identified in this study was the PI3K-AKT pathway, and 12 related genes, including IGF1R, FGF9, PDGFRA, GYS1, PKN2, CREB3L1, TLR4, TNN, EFNA4, BCL2L1, LAMC1, and GNG12, were predicted as targets of miR-140, implying that miR-140 can negatively regulate the functions of the PI3K-AKT pathway and its downstream biological processes. The PI3K-AKT pathway regulates a variety of biological processes, including inflammation, cell growth, survival, and metabolism, in response to growth factors and has also been reported to be involved in both OA pathogenesis and cellular senescence.[43,44] For example, activation of the PI3K-AKT pathway in chondrocytes promoted the expression of collagenolytic MMPs and ultimately accelerated hypertrophy and degradation of the ECM, whereas inhibition of the PI3K-AKT pathway attenuated cartilage degradation and the inflammatory response.[45,46] Various stresses, such as IL-1β stimulation and trauma, can induce chondrocyte senescence, primarily through activating the PI3K-AKT pathway and subsequently upregulating p53 and p21 expression, which in turn, stimulates SASP.[47] In addition, DDR activates the checkpoint kinases ATM/ATR and CHK1/2, leading to p53 accumulation and activation through which persistent DNA damage foci result in DNA segments with chromatin alterations that reinforce cell senescence (DNA SCARS); DDR also activates GATA4, which serves as a critical regulator of nuclear factor κB (NF-κB) pathway activation and subsequent SASP activation.[27] Moreover, activation of PI3K-AKT can also trigger the NF-κB pathway in chondrocytes.[46] Therefore, the inhibition of chondrocyte senescence by miR-140, found in this study, may also be partially achieved through the PI3K-AKT pathway (Figure 6).

We found both in human and rat cartilage that the senescence-related markers, expressed mainly at the superficial layer of AC in E-OA and the related SnCC localization, might have important relevance for blocking cartilage regeneration, as cartilage progenitor cells (CPCs), a subtype of chondrocytes with potential stem cell properties, are mainly located in this region.[48,49] Notch and PI3K-AKT pathway-related molecules are expressed in both mesenchymal stem cells (MSCs) and CPCs,[39,42,43] and activation of the Notch pathway, especially by the JAG1-mediated route, significantly inhibits chondrogenic differentiation of MSCs by regulation of cellular senescence.[42,50,51] PI3K-AKT pathway activation can also promote MSC senescence.[44] These results suggest that miR-140 may promote OA cartilage damage repair by regulating Notch and PI3K-AKT pathway-mediated CPC senescence, although this possibility requires further validation. Moreover, there is a close interaction between Notch and PI3K-AKT signaling, and these pathways are involved in a wide variety of cellular processes. For example, Guo et al.[52] reported that Notch2 may negatively regulate cell invasion by inhibiting the PI3K-AKT signaling pathway in gastric cancer, and Villegas et al.[53] found that PI3K-AKT cooperates with oncogenic Notch by inducing nitric oxide-dependent inflammation. However, few studies have reported the interaction between the Notch and PI3K-AKT pathways in chondrocytes, and further research on this issue may further enrich the theory of OA pathogenesis and promote the exploration of new OA therapeutic targets. Of course, it should be noted that the regulation of Notch and PI3K-AKT signaling in OA remains controversial.[33] For example, Hosaka et al.[40] reported that activation of Notch signaling in chondrocytes contributes to the development of OA, whereas Liu et al.[54] showed that inhibition of Notch signaling in chondrocytes results in a progressive OA-like pathology. Lim et al.[55] reported that inhibition of PI3K-AKT attenuated cartilage degradation, whereas Kita et al.[56] and Rao et al.[57] found that PI3K-AKT activation protects chondrocytes from apoptosis due to endoplasmic reticulum stress and that it is essential for chondrocyte proliferation. Based on existing research, it is difficult to interpret these contradictory results, and therefore, further studies on the roles of the Notch and PI3K-AKT pathways in OA pathogenesis are necessary.

Chondrocytes are solely responsible for the synthesis of ECM components.[5,58] However, cartilage cellularity is reduced in OA, and the remaining chondrocytes are activated by various cytokines to express a catabolic and abnormal phenotype with poor proliferation and migration potential, resulting in accelerated degradation and limited capacity of the ECM for self-repair and regeneration.[3–5,21] In the development of OA, SnCCs secrete various proinflammatory cytokines, chemokines, catabolic enzymes, and other factors and further derange the chondrocyte phenotype toward a hypertrophic phenotype, shifting the balance between synthesis and degradation of ECM in favor of catabolic events.[3,4,21] MMP13 and ADAMTS5 are well-known proteases that can degrade a wide range of ECM components, especially COL2 and ACAN, which are the major structural components of the AC and provide tensile strength and shock absorption under mechanical damage.[16,17] Therefore, agents targeting MMP13 and ADAMTS5 will help promote ECM homeostasis.

Previous studies have shown that both MMP13 and ADAMTS5 are targets of miR-140, and the expression of MMP13 and ADAMTS5 can be inhibited, whereas COL2 can be promoted by miR-140 in natural OA cartilage-derived chondrocytes.[20,36] We further reported in this study that miR-140 could partially counteract IL-1β- and trauma-induced expression of MMP13 and ADAMTS5 and degradation of COL2 and ACAN, suggesting that miR-140 can stabilize ECM homeostasis in a variety of OA environments.

Intra-articular delivery of drugs or genes offers the ability to sustain therapeutically effective concentrations and provide a secure environment with fewer extra-articular adverse effects.[47,59] According to most previous studies, the local delivery of genes is dependent on bioactive carriers, such as atelocollagen, lentiviruses, and self-complementary adeno-associated viruses (scAAVs).[60–62] However, bioactive carriers may have biotoxic effects,[63] and the direct modification of known drugs is a widely used strategy for prolonging their residence time and reducing biotoxicity.[59] In this study, the miR-140 used *in vivo* was a chemically modified miR agonist, as reported previously (Figure S6B).[36,64] It could rapidly enter the cartilage and reach the chondrocyte cytoplasm after IAJ without extra-articular uptake and then delay chondrocyte senescence and OA progression without inducing complications. These findings suggest that chemically modified miR-140 is a promising therapeutic agent for OA and lay the foundation for the development of miR-based OA therapeutic strategies. Additionally, OA is a whole joint disease rather than a dysfunction of merely cartilage. Peng et al.[61] reported that synovial fibroblasts (SFs) are responsive to miR-140 and that they could inhibit SF proliferation and migration and promote SF apoptosis in autoimmune arthritis mice. In addition, Waki et al.[65] and Li et al.[66] reported that miR-140 played important roles in fracture healing, and Genemaras et al.[67] reported that miR-140 could be detected in meniscus cells, but they did not study the effects of miR-140 on osteoblasts, osteoclasts, or meniscus cells. Therefore, the effects of miR-140 on cells and tissues other than chondrocytes and cartilage, as well as cartilage-targeted delivery strategies, should be further investigated.

There are several limitations to this study. First, the expression of senescence-related markers, particularly $p16^{INK4a}$, is well known to be age dependent.[68] The donors in the OA groups were relatively older than those in the normal group, although the difference did not reach statistical significance (Table S1) in this study. Second, although IL-1β stimulation and surgical induction (ACLT + DMM) are classical and widely used methods to mimic OA-like changes,[69,70] OA pathology is much more complex, and an miR-140 knockout mouse model may be more helpful in studying the related molecular mechanisms. Furthermore, we only used male rats to mimic *in vivo* OA. Therefore, it is necessary to verify these results in female rats in subsequent studies because estrogen may increase miR-140 expression to protect cartilage from degradation.[71] Third, we focused mainly on the effects of miR-140 on chondrocytes and cartilage but did not investigate the effects of miR-140 on the senescent features of other cells (such as fibroblasts, osteoblasts, osteoclasts, and meniscus cells) and tissues (such as synovium, meniscus, and subchondral bone) in the joint. For example, previous studies, such as those reported by Jeon et al.[18] and Farr,[72] have established a causal role of senescent cells in bone loss and have indicated that the targeting of these cells has antiresorptive, anabolic, and remodeling effects on bone; thus, there is an extensive need for further research on the effects of miR-140 in the joint. Finally, we injected only a single dose of miR-140 at the early stage of OA, and an investigation of the therapeutic effects of multiple injections of miR-140 at different time intervals on OA progression is currently under way in our group. We hope to report more information on this topic in the near future.

Taken together, this study provides new evidence that chondrocyte senescence correlates with OA pathogenesis and that miR-140 can effectively attenuate the progression of E-OA by retarding chondrocyte senescence and stabilizing ECM homeostasis. Our study contributes new evidence of the involvement of miR-mediated epigenetic regulation of chondrocyte senescence in OA pathogenesis. Additional comprehensive studies are required to investigate the underlying mechanisms and to explore the potential targets for OA therapeutics.

MATERIALS AND METHODS

Human Cartilage Specimens

This study was approved by the Institutional Ethics Committee of West China Hospital, Sichuan University, and written informed consent was obtained from all of the donors. Knee OA was diagnosed based on clinical and radiological evaluation, and its severity was classified according to the Kellgren and Lawrence (KL) X-ray criteria.[73] Normal cartilage specimens were collected from trauma amputees without any history of OA or other knee diseases, and E-OA (KL grade 1–2) and ML-OA (KL grade 3–4) cartilage specimens were collected from patients with primary OA undergoing knee arthroplasty at the Orthopaedics of West China Hospital, Sichuan University (n = 5 in each group). All cartilage was dissected from the medial femoral condyles, and the OA specimens were taken from the area around the cartilage lesions (within 1.5 cm). The mean ages of the donors with normal, E-OA, and ML-OA knees were 48.00 (39–58), 57.80 (51–65), and 58.60 (51–63) years, respectively, and additional characteristics of the donors are shown in Table S1.

Isolation and Culture of Primary Human Articular Chondrocytes

A part of each cartilage was used to isolate chondrocytes, as described previously,[36] and the remainder was used for histologic and immunohistochemical analyses. For chondrocyte isolation, the cartilage was washed, minced, sequentially digested for 15 min with 0.25% trypsin (HyClone, Logan, UT, USA) and for 4–8 h with 0.2% (2 mg/mL) COL2 (Sigma, St. Louis, MO, USA) on an orbital shaker at 37°C, and filtered through a 100-μm cell strainer (BD-Falcon, Franklin Lakes, NJ, USA). After washing with high glucose DMEM (DMEM-HG; HyClone) containing 1% penicillin/streptomycin (HyClone), the freshly isolated chondrocytes (defined as passage 0 [P0]) were resuspended in DMEM-HG, supplemented with 15% fetal bovine serum (BI Beit-Haemek, Western Galilee, IL) and 1% penicillin/streptomycin, seeded at a density of 4×10^4 cells/cm^2,

and incubated with 5% CO_2 at 37°C. The media were replaced 24 h after seeding to remove the nonadherent cells and then replaced every 2–3 days. When the monolayer culture reached 80% ~90% confluence, one flask or plate was lysed directly for RNA extraction, whereas the cells in the other plates were detached by trypsin and reseeded at 2×10^4 cells/cm^2 (P1) for use in subsequent experiments.

In Vitro IL-1β Stimulation and miR-140 Transfection

In vitro chondrocyte senescence was induced by 24-h IL-1β (MCE, Pudong, Shanghai, China) stimulation of normal chondrocytes.[22] A time course of SA-βGal activity and p16^{INK4a} staining, including time points of 0 h, 24 h, 48 h, 72 h, 5 days, and 7 days after IL-1β treatment at different concentrations (1, 2, 5, and 10 ng/mL), was investigated, and the IL-1β stimulation paradigm was optimized. A 24-h, 5 ng/mL IL-1β treatment was selected for *in vitro* chondrocyte senescence establishment, and a further 48-h incubation in fresh media was allowed before the final analysis (Figures S2A and S2B). In the experiments using miR-140 mimic (50 nM; RiboBio, Guangzhou, Guangdong, China) or miR-140 negative control (50 nM, scrambled 22 nt with no homology to mammal genome, miR-Scr; RiboBio), transfection was performed using a *FECT* CP Transfection Kit (RiboBio), following the manufacturer's protocols, 24 h before other experimental procedures, and the efficiency of transfection was monitored by quantitative RT-PCR, 24 h after transfection. To distinguish senescent and dysfunctional cells, the IL-1β-treated chondrocytes were also stimulated with FGF2 (1 ng/mL and 10 ng/mL; MCE) and incubated for 48 h for cell cycle analysis.

SA-βGal Assay

SA-βGal activity was measured using a staining kit (Beyotime, Nantong, Jiangsu, China) and a mammalian SA-βGal assay kit (Thermo Fisher Scientific, Waltham, MA, USA), according to the manufacturer's protocols. For SA-βGal staining, chondrocytes were plated and coverslipped, fixed for 15 min at room temperature, washed, and incubated with the staining solution overnight at 37°C. Coverslips were photographed with a Zeiss microscope (Oberkochen, Germany), and the percentage of SA-βGal-positive cells from three random fields was quantified and averaged using the Zeiss Application Suite at a magnification of 400× . For SA-βGal activity, chondrocytes were plated and intervened in 96-well plates. After washing, 100 μL of β-Galactosidase Assay Reagent was added, the cells were incubated for 30 min at 37°C, and the absorbance was measured at 405 nm.

Real-Time PCR

Total RNA was extracted using TRIzol Reagent (Invitrogen, Carlsbad, CA, USA) and reverse transcribed to first-strand cDNA using a TaqMan Reverse Transcription Kit, according to the manufacturer's protocols (Applied Biosystems, Carlsbad, CA, USA). Primers were synthesized by Sangon (Shanghai, China) (Table S2), and PCR assays were performed in triplicate using Real-Time PCRBIO SyGreen Mix and an ABI7900 PCR system (Applied Biosystems) and averaged. For each sample, differences in the threshold cycle (ΔCt) values were calculated by correcting the Ct of the target genes to the Ct of the reference gene *β-actin* (*ACTB*), and the relative gene expression was expressed as $2^{-\Delta\Delta Ct}$ with respect to the control group.[74] For miR-140 quantification, MicroRNA Purification Kit (Norgen, Thorold, ON, CA) and Bulge-loop miRNA qRT-PCR Primer Sets (one reverse transcriptase primer and a pair of qPCR primers for each set) specific for miR-140 (RiboBio) were used, according to the manufacturer's protocols, and small nuclear RNA (snRNA) U6 was utilized as an internal control.

Western Blotting

Total protein was extracted using radioimmunoprecipitation assay (RIPA) buffer (Beyotime) with an enzyme inhibitor cocktail. After being lysed on ice for 60 min, the lysate was centrifuged, and the supernatant was collected. The concentration of the extracted protein was determined using a Bicinchoninic Acid (BCA) Protein Assay Kit (Pierce, Rockford, IL, USA), and the remaining protein was mixed with 4 times loading dye (Pierce) and heated at 100°C for 10 min. Equal amounts of proteins were separated using SDS-PAGE and transferred to polyvinylidene difluoride membranes (Hybond, Piscataway, NJ, USA). Following blocking in 5% skim milk for 60 min, the membranes were incubated at 4°C overnight with primary antibodies, including anti-p16^{INK4a} (1:200; Abcam, Cambridge, MA, USA; Cat. No: ab108349); anti-p21 (1:200; Bioss, Beijing, China; Cat. No: bs-10129R); anti-p53 (1:500; GenXspan, AL, USA; Cat. No: GXP509294); anti-COL2 (1:1,000; Abcam, Cambridge, MA, USA; Cat. No: ab34710); anti-ACAN (1:500; Novus, Littleton, CO, USA; Cat. No: CS-56); anti-MMP13 (1:500; ABclonal, Boston, MA, USA; Cat. No: A2427); anti-ADAMTS5 (1:500; Novus; Cat. No: NBP2-15286); anti-β-actin (1:2,000; Santa, Dallas, TX, USA; Cat. No: AC002); and anti-glyceraldehyde 3-phosphate dehydrogenase (GAPDH; 1:2,000; ABclonal; Cat. No: TA-08). The membranes were then incubated with secondary antibodies at 37°C for 1 h and visualized using an enhanced chemiluminescence solution (Amersham, Chiltern, Buckinghamshire, UK). The gray values of the bands were quantitatively analyzed using ImageJ software.

Immunocytochemistry

Chondrocytes were inoculated in 6-well plates at a density of 1×10^4 cells/cm^2 with sterile coverslips and cultured prior to intervention. At specific time points, the coverslips were removed, the culture was rinsed, and the cells were fixed with 10% paraformaldehyde for 30 min at room temperature. After washing, the cells were permeabilized using 0.1% Triton X-100 for 10 min and then washed. Afterward, the cells were incubated with 3% H_2O_2 at room temperature for 10 min and washed and blocked with blocking solution at room temperature for 30 min. The cells were rinsed and incubated with primary rabbit antibodies directed against human γH2AX (1:400; CST, Danvers, MA, USA; Cat. No: 9718) and p16^{INK4a} (1:100; Abcam), followed by washing again. Finally, the cells were incubated with secondary antibodies and then with chromogen substrate. The γH2AX-positive cells, characterized by dot-like intranuclear puncta, and p16^{INK4a}-positive cells were manually counted using ImageJ software (NIH, Bethesda, MD, USA), and three random microscopic fields (400 times; Zeiss) were selected and averaged for each coverslip. All

of the coverslips were assessed independently by three blinded assessors (H.-b.S., L.L., and M.T.), and the final results were determined via a consensus.

Flow Cytometric Cell Cycle Analysis

After trypsin treatment, detached chondrocytes were fixed with 70% ice-cold ethanol overnight at 4°C. The cells were digested with 100 μg/mL RNase at 37°C for 20 min and then stained with 50 μg/mL propidium iodide for 30 min in the dark. Filtered samples were then analyzed for cycle content on a Celula Sparrow cell sorter, using Celula AISFCM Analyze software (Huaxi Technology Park, Chengdu, Sichuan, China), and the percentage of cells in the G0/G1, S, and G2/M phases was determined using ModFit LT software (Verity Software House, Topsham, ME, USA).

In Vivo OA Model Establishment

Twelve-week-old male Sprague-Dawley rats (250 ± 20 g) were purchased and housed at the Laboratory Animal Centre of Sichuan University under standard diurnal light/dark conditions, fed a standard commercial diet, and allowed access to tap water *ad libitum*. All animals received humane care, and all procedures were carried out according to the Guide for the Care and Use of Laboratory Animals. The rats were anesthetized by intraperitoneal injection of pentobarbital sodium (40 mg/kg; Tocris, Bristol, UK), and OA was surgically induced in the right hind knee by ACLT + DMM, according to previous reports.[36,70] All surgical procedures were performed under sterile conditions, and the rats were allowed to move, eat, and drink freely after surgery.

IAJ of miR-140 and Fluorescence Distribution Imaging

The miR-140 agomir and miR agomir negative control (scrambled 22 nt, miR-Scr; RiboBio) solutions were prepared immediately before injection. One week after surgery, once the wound healed, IAJ was performed under ultrasonic guidance through a medial patellar approach using an insulin syringe with a 29G needle (BD, New York, NY, USA), and then, the rats were allowed unrestricted weight bearing and motion. For *in vivo* fluorescence imaging, 5 model rats were treated with equal amounts of Cy5-labeled miR-140 agomir (5 nmol in 100 μL double-distilled H_2O [ddH_2O]), administered to the right hind knee, whereas NS was administered to the left hind knee. Twenty-four hours later, the rats were anesthetized, and fluorescence was measured using a SpectrumCT *in vivo* imaging system (PerkinElmer, Waltham, MA, USA). Then, the rats were sacrificed, and the bilateral femoral condyles in the hind knees were obtained. After washing, the condyles were coated with optimal cutting temperature compound (OCT) and quickly frozen, and 6-μm sections were prepared using a Leica RM2235 manual rotary microtome (Wetzlar, Hessen, Germany) and a Feather microtome blade (Osaka, Japan) and naturally dried away from light. Fluorescent images of the sections were acquired by a fluorescence microscope (Zeiss). For subsequent experiments, 30 model rats were randomized into miR-Scr and miR-140 groups (n = 15 in each) and intra-articularly injected with equal amounts (5 nmol in 100 μL ddH_2O) of miR-Scr and miR-140 agomir, respectively. Five normal rats were designated as normal controls.

Behavioral and Macroscopic Evaluation

At 2, 4, and 6 weeks after modeling surgery (T1, T2, and T3, respectively), 5 rats in each group were randomly selected for evaluation of behavioral changes, as previously described.[75,76] Briefly, each rat touched the white paper-covered walls of an open field box with its forelimbs, which were stained with ink from foam stamp pads that covered the floor of the box. The rats were monitored for a 25-min period, and the number of rears above a point 5 cm from the button of the paper was counted. After the behavioral evaluation, the rats were sacrificed, and the femoral condyles were harvested. The gross morphology of the cartilage was rapidly photographed and scored using the following 0–4 scale: 0 = surface appears normal; 1 = minimal fibrillation or slight yellowish discoloration of the surface; 2 = erosion extending to the superficial or middle layers; 3 = erosion extending to the deep layer; and 4 = erosion extending to the subchondral bone.[77,78] Then, the condyles were used for further histological and immunohistochemical analyses.

Histologic and Immunohistochemical Analyses

The human and rat cartilage tissues were routinely fixed with 4% paraformaldehyde at 4°C for 48 h and then decalcified with 10% EDTA at pH 7.4 for 4–6 weeks. After dehydration, all tissues were embedded in paraffin and sagittally sectioned at 6 μm thickness. The sections were de-waxed and hydrated before being stained with H&E and Safranin O. The severity of the cartilage lesions in the loading area of the medial femoral condyle was graded using the modified Mankin scoring system. The chondrocyte number in an equal area (400 × 200 μm, from the surface to the deep zone) and the cartilage thickness (from the surface to the deep zone) were also measured as reported previously.[79] For immunohistochemistry, the paraffin-embedded sections were processed and incubated with diluted antibodies of $p16^{INK4a}$ (1:100; Bioss, Cat. No: bs-0740R); p21 (1:100; Bioss); p53 (1:100; GenXspan); γH2AX (1:200; CST); COL2 (1:200; Abcam); ACAN (1:100; Novus); MMP13 (1:100; ABclonal); and ADAMTS5 (1:100; Novus) before staining with secondary antibodies and chromogen substrate. The percentage of immunopositively stained chondrocytes was determined using ImageJ software, and three random microscopic fields (400 times; Zeiss) in the loading area were selected and averaged in each section. All sections were assessed independently by three blinded assessors (H.-b.S., L.L., and M.T.), and the final results were determined via a consensus.

Bioinformatics Analysis of the Possible Mechanism of miR-140-5p

A bioinformatics analysis was further conducted to assess the potential mechanism of miR-140. The mature sequence of hsa-miR-140-5p was obtained from the NCBI and University of California, Santa Cruz (UCSC), databases, and the putative targets of hsa-miR-140-5p were determined using the miRDB, miRmap, PicTar, TargetScan (Release 7.2), and DIANA microT-CDS databases. To make the predicted target genes more convincible, only the target genes predicted by at least three databases were selected for further analysis. The relatedness of these target genes in cellular networks was investigated by GO

analysis using the Database for Annotation, Visualization and Integrated Discovery (DAVID; version 6.8). The probable signaling pathways in which these target genes were enriched were analyzed by the KEGG database, and $p < 0.05$ was considered statistically significant. Finally, the interactive relationships among the proteins encoded by the target genes were mapped using the STRING database (version 11.0), and only the interactions with a combined score >0.4 were considered significant. To validate the effect of miR-140 on the proteins encoded by the predicted target genes of hsa-miR-140-5p, normal human cartilage-derived chondrocytes were used, and IL-1β stimulation (24 h, 5 ng/mL), miR-Scr/miR-140 transfection, and western blotting were conducted as described before. The primary antibodies, including anti-JAG1 (1:1,000; Invitrogen; Cat. No: PA5-72843), anti-NUMBL (1:500; Affinity, Cincinnati, OH, USA; Cat. No: DF6863), anti-IGF1R (1:1,000; Abcam; Cat. No: ab131476), and anti-TLR4 (1:500; Abcam; Cat. No: ab13556), were used.

Statistical Analysis

All experiments were performed using samples from at least three donors or rats. All quantitative data were presented as the mean and 95% confidence intervals (CIs) and assessed for normality of distribution using the Kolmogorov-Smirnov and Shapiro-Wilk tests before data analysis. The t test or the Mann-Whitney *U* test was used to identify the significance of the differences between two groups, whereas one-way ANOVA with Tukey's post hoc analysis or Kruskal-Wallis *H* with Student-Newman-Keuls (SNK) post hoc analysis was carried out for multiple group comparisons. All statistical analyses were performed using SPSS software (version 22.0; IBM, Armonk, NY, USA), and p values <0.05 were considered statistically significant.

SUPPLEMENTAL INFORMATION

Supplemental Information can be found online at https://doi.org/10.1016/j.omtn.2019.10.032.

AUTHOR CONTRIBUTIONS

H.-b.S. and B.S. conceived the project and designed the experiments. H.-b.S., T.-m.Y., P.-d.K., J.Y., Z.-k.Z., and B.S. screened and collected the human tissues. H.-b.S., L.L., M.T., L.Z., and D.-p.L. performed the experiments. H.-b.S., L.L., and B.S. acquired, analyzed, and interpreted the data. H.-b.S. and B.S. wrote the manuscript. H.-b.S., J.-q.C., and B.S. supervised the project. All authors approved the final manuscript.

CONFLICTS OF INTEREST

The authors declare no competing interests.

ACKNOWLEDGMENTS

The work was done in Chengdu, Sichuan Province, China. This study was supported through grants from the National Natural Science Foundation of China (81802210, 81672219, and 81974347); Key Project of Sichuan Science & Technology Department (2018SZ0223 and 2018SZ0250); China Postdoctoral Science Foundation (2017M610604); and National Clinical Research Center for Geriatrics, West China Hospital, Sichuan University (Z2018B20).

REFERENCES

1. Loeser, R.F., Collins, J.A., and Diekman, B.O. (2016). Ageing and the pathogenesis of osteoarthritis. Nat. Rev. Rheumatol. *12*, 412–420.
2. Hunter, D.J., and Bierma-Zeinstra, S. (2019). Osteoarthritis. Lancet *393*, 1745–1759.
3. Rahmati, M., Nalesso, G., Mobasheri, A., and Mozafari, M. (2017). Aging and osteoarthritis: Central role of the extracellular matrix. Ageing Res. Rev. *40*, 20–30.
4. McCulloch, K., Litherland, G.J., and Rai, T.S. (2017). Cellular senescence in osteoarthritis pathology. Aging Cell *16*, 210–218.
5. Glyn-Jones, S., Palmer, A.J., Agricola, R., Price, A.J., Vincent, T.L., Weinans, H., and Carr, A.J. (2015). Osteoarthritis. Lancet *386*, 376–387.
6. Poulet, B., and Staines, K.A. (2016). New developments in osteoarthritis and cartilage biology. Curr. Opin. Pharmacol. *28*, 8–13.
7. Xu, M., Bradley, E.W., Weivoda, M.M., Hwang, S.M., Pirtskhalava, T., Decklever, T., Curran, G.L., Ogrodnik, M., Jurk, D., Johnson, K.O., et al. (2017). Transplanted senescent cells induce an osteoarthritis-like condition in mice. J. Gerontol. A Biol. Sci. Med. Sci. *72*, 780–785.
8. Toh, W.S., Brittberg, M., Farr, J., Foldager, C.B., Gomoll, A.H., Hui, J.H., Richardson, J.B., Roberts, S., and Spector, M. (2016). Cellular senescence in aging and osteoarthritis. Acta Orthop. *87* (sup363), 6–14.
9. Price, J.S., Waters, J.G., Darrah, C., Pennington, C., Edwards, D.R., Donell, S.T., and Clark, I.M. (2002). The role of chondrocyte senescence in osteoarthritis. Aging Cell *1*, 57–65.
10. Platas, J., Guillén, M.I., Pérez Del Caz, M.D., Gomar, F., Castejón, M.A., Mirabet, V., and Alcaraz, M.J. (2016). Paracrine effects of human adipose-derived mesenchymal stem cells in inflammatory stress-induced senescence features of osteoarthritic chondrocytes. Aging (Albany N.Y.) *8*, 1703–1717.
11. Ashraf, S., Cha, B.H., Kim, J.S., Ahn, J., Han, I., Park, H., and Lee, S.H. (2016). Regulation of senescence associated signaling mechanisms in chondrocytes for cartilage tissue regeneration. Osteoarthritis Cartilage *24*, 196–205.
12. Zhang, D., Zhang, G., Li, Z., and Li, B. (2018). Activation of the cannabinoid receptor 1 by ACEA suppresses senescence in human primary chondrocytes through sirt1 activation. Exp. Biol. Med. (Maywood) *243*, 437–443.
13. Dai, S.M., Shan, Z.Z., Nakamura, H., Masuko-Hongo, K., Kato, T., Nishioka, K., and Yudoh, K. (2006). Catabolic stress induces features of chondrocyte senescence through overexpression of caveolin 1: possible involvement of caveolin 1-induced down-regulation of articular chondrocytes in the pathogenesis of osteoarthritis. Arthritis Rheum. *54*, 818–831.
14. Kapoor, M., Martel-Pelletier, J., Lajeunesse, D., Pelletier, J.P., and Fahmi, H. (2011). Role of proinflammatory cytokines in the pathophysiology of osteoarthritis. Nat. Rev. Rheumatol. *7*, 33–42.
15. Liu, Y., Zhu, H., Yan, X., Gu, H., Gu, Z., and Liu, F. (2017). Endoplasmic reticulum stress participates in the progress of senescence and apoptosis of osteoarthritis chondrocytes. Biochem. Biophys. Res. Commun. *491*, 368–373.
16. Fosang, A.J., Last, K., Knäuper, V., Murphy, G., and Neame, P.J. (1996). Degradation of cartilage aggrecan by collagenase-3 (MMP-13). FEBS Lett. *380*, 17–20.
17. Goldring, M.B., and Marcu, K.B. (2009). Cartilage homeostasis in health and rheumatic diseases. Arthritis Res. Ther. *11*, 224.
18. Jeon, O.H., Kim, C., Laberge, R.M., Demaria, M., Rathod, S., Vasserot, A.P., Chung, J.W., Kim, D.H., Poon, Y., David, N., et al. (2017). Local clearance of senescent cells attenuates the development of post-traumatic osteoarthritis and creates a pro-regenerative environment. Nat. Med. *23*, 775–781.
19. Papaioannou, G., Mirzamohammadi, F., Lisse, T.S., Nishimori, S., Wein, M.N., and Kobayashi, T. (2015). MicroRNA-140 provides robustness to the regulation of hypertrophic chondrocyte differentiation by the PTHrP-HDAC4 pathway. J. Bone Miner. Res. *30*, 1044–1052.
20. Zhang, R., Ma, J., and Yao, J. (2013). Molecular mechanisms of the cartilage-specific microRNA-140 in osteoarthritis. Inflamm. Res. *62*, 871–877.
21. Miyaki, S., Nakasa, T., Otsuki, S., Grogan, S.P., Higashiyama, R., Inoue, A., Kato, Y., Sato, T., Lotz, M.K., and Asahara, H. (2009). MicroRNA-140 is expressed in differentiated human articular chondrocytes and modulates interleukin-1 responses. Arthritis Rheum. *60*, 2723–2730.

22. Karlsen, T.A., de Souza, G.A., Ødegaard, B., Engebretsen, L., and Brinchmann, J.E. (2016). microRNA-140 Inhibits Inflammation and Stimulates Chondrogenesis in a Model of Interleukin 1β-induced Osteoarthritis. Mol. Ther. Nucleic Acids *5*, e373.

23. Si, H., Zeng, Y., Zhou, Z., Pei, F., Lu, Y., Cheng, J., and Shen, B. (2016). Expression of miRNA-140 in chondrocytes and synovial fluid of knee Joints in patients with osteoarthritis. Chin. Med. Sci. J. *31*, 207–212.

24. Nugent, M. (2016). MicroRNAs: exploring new horizons in osteoarthritis. Osteoarthritis Cartilage *24*, 573–580.

25. Dobson, G.P., Letson, H.L., Grant, A., McEwen, P., Hazratwala, K., Wilkinson, M., and Morris, J.L. (2018). Defining the osteoarthritis patient: back to the future. Osteoarthritis Cartilage *26*, 1003–1007.

26. Gao, S.G., Zeng, C., Li, L.J., Luo, W., Zhang, F.J., Tian, J., Cheng, C., Tu, M., Xiong, Y.L., Jiang, W., et al. (2016). Correlation between senescence-associated beta-galactosidase expression in articular cartilage and disease severity of patients with knee osteoarthritis. Int. J. Rheum. Dis. *19*, 226–232.

27. Pignolo, R.J., Samsonraj, R.M., Law, S.F., Wang, H., and Chandra, A. (2019). Targeting Cell Senescence for the Treatment of Age-Related Bone Loss. Curr. Osteoporos. Rep. *17*, 70–85.

28. Hall, B.M., Balan, V., Gleiberman, A.S., Strom, E., Krasnov, P., Virtuoso, L.P., Rydkina, E., Vujcic, S., Balan, K., Gitlin, I., et al. (2016). Aging of mice is associated with p16(Ink4a)- and β-galactosidase-positive macrophage accumulation that can be induced in young mice by senescent cells. Aging (Albany N.Y.) *8*, 1294–1315.

29. Stein, G.H., Drullinger, L.F., Soulard, A., and Dulić, V. (1999). Differential roles for cyclin-dependent kinase inhibitors p21 and p16 in the mechanisms of senescence and differentiation in human fibroblasts. Mol. Cell. Biol. *19*, 2109–2117.

30. Martin, J.A., and Buckwalter, J.A. (2003). The role of chondrocyte senescence in the pathogenesis of osteoarthritis and in limiting cartilage repair. J. Bone Joint Surg. Am. *85-A* (*Suppl 2*), 106–110.

31. Aigner, T., Hemmel, M., Neureiter, D., Gebhard, P.M., Zeiler, G., Kirchner, T., and McKenna, L. (2001). Apoptotic cell death is not a widespread phenomenon in normal aging and osteoarthritis human articular knee cartilage: a study of proliferation, programmed cell death (apoptosis), and viability of chondrocytes in normal and osteoarthritic human knee cartilage. Arthritis Rheum. *44*, 1304–1312.

32. Rodier, F., and Campisi, J. (2011). Four faces of cellular senescence. J. Cell Biol. *192*, 547–556.

33. Shane Anderson, A., and Loeser, R.F. (2010). Why is osteoarthritis an age-related disease? Best Pract. Res. Clin. Rheumatol. *24*, 15–26.

34. Greene, M.A., and Loeser, R.F. (2015). Aging-related inflammation in osteoarthritis. Osteoarthritis Cartilage *23*, 1966–1971.

35. Miyaki, S., and Asahara, H. (2012). Macro view of microRNA function in osteoarthritis. Nat. Rev. Rheumatol. *8*, 543–552.

36. Si, H.B., Zeng, Y., Liu, S.Y., Zhou, Z.K., Chen, Y.N., Cheng, J.Q., Lu, Y.R., and Shen, B. (2017). Intra-articular injection of microRNA-140 (miRNA-140) alleviates osteoarthritis (OA) progression by modulating extracellular matrix (ECM) homeostasis in rats. Osteoarthritis Cartilage *25*, 1698–1707.

37. Saito, T., and Tanaka, S. (2017). Molecular mechanisms underlying osteoarthritis development: Notch and NF-κB. Arthritis Res. Ther. *19*, 94.

38. Hoare, M., and Narita, M. (2018). Notch and Senescence. Adv. Exp. Med. Biol. *1066*, 299–318.

39. Kagawa, S., Natsuizaka, M., Whelan, K.A., Facompre, N., Naganuma, S., Ohashi, S., Kinugasa, H., Egloff, A.M., Basu, D., Gimotty, P.A., et al. (2015). Cellular senescence checkpoint function determines differential Notch1-dependent oncogenic and tumor-suppressor activities. Oncogene *34*, 2347–2359.

40. Hosaka, Y., Saito, T., Sugita, S., Hikata, T., Kobayashi, H., Fukai, A., Taniguchi, Y., Hirata, M., Akiyama, H., Chung, U.I., and Kawaguchi, H. (2013). Notch signaling in chondrocytes modulates endochondral ossification and osteoarthritis development. Proc. Natl. Acad. Sci. USA *110*, 1875–1880.

41. Sugita, S., Hosaka, Y., Okada, K., Mori, D., Yano, F., Kobayashi, H., Taniguchi, Y., Mori, Y., Okuma, T., Chang, S.H., et al. (2015). Transcription factor Hes1 modulates osteoarthritis development in cooperation with calcium/calmodulin-dependent protein kinase 2. Proc. Natl. Acad. Sci. USA *112*, 3080–3085.

42. Li, F., Shi, W., Wan, Y., Wang, Q., Feng, W., Yan, X., Wang, J., Chai, L., Zhang, Q., and Li, M. (2017). Prediction of target genes for miR-140-5p in pulmonary arterial hypertension using bioinformatics methods. FEBS Open Bio *7*, 1880–1890.

43. Xie, L., Xie, H., Chen, C., Tao, Z., Zhang, C., and Cai, L. (2019). Inhibiting the PI3K/AKT/NF-κB signal pathway with nobiletin for attenuating the development of osteoarthritis: in vitro and in vivo studies. Food Funct. *10*, 2161–2175.

44. Chen, H., Shi, B., Feng, X., Kong, W., Chen, W., Geng, L., Chen, J., Liu, R., Li, X., Chen, W., et al. (2015). Leptin and Neutrophil-Activating Peptide 2 Promote Mesenchymal Stem Cell Senescence Through Activation of the Phosphatidylinositol 3-Kinase/Akt Pathway in Patients With Systemic Lupus Erythematosus. Arthritis Rheumatol. *67*, 2383–2393.

45. Fu, D., Shang, X., Ni, Z., and Shi, G. (2016). Shikonin inhibits inflammation and chondrocyte apoptosis by regulation of the PI3K/Akt signaling pathway in a rat model of osteoarthritis. Exp. Ther. Med. *12*, 2735–2740.

46. Xue, J.F., Shi, Z.M., Zou, J., and Li, X.L. (2017). Inhibition of PI3K/AKT/mTOR signaling pathway promotes autophagy of articular chondrocytes and attenuates inflammatory response in rats with osteoarthritis. Biomed. Pharmacother. *89*, 1252–1261.

47. Li, T.F., Gao, L., Sheu, T.J., Sampson, E.R., Flick, L.M., Konttinen, Y.T., Chen, D., Schwarz, E.M., Zuscik, M.J., Jonason, J.H., and O'Keefe, R.J. (2010). Aberrant hypertrophy in Smad3-deficient murine chondrocytes is rescued by restoring transforming growth factor beta-activated kinase 1/activating transcription factor 2 signaling: a potential clinical implication for osteoarthritis. Arthritis Rheum. *62*, 2359–2369.

48. McGonagle, D., Baboolal, T.G., and Jones, E. (2017). Native joint-resident mesenchymal stem cells for cartilage repair in osteoarthritis. Nat. Rev. Rheumatol. *13*, 719–730.

49. Si, H., Liang, M., Cheng, J., and Shen, B. (2019). [Effects of cartilage progenitor cells and microRNA-140 on repair of osteoarthritic cartilage injury]. Zhongguo Xiu Fu Chong Jian Wai Ke Za Zhi *33*, 650–658.

50. Cao, Z., Liu, C., Bai, Y., Dou, C., Li, J.M., Shi, D.W., Dong, S.W., and Xiang, Q. (2017). Inhibitory effect of dihydroartemisinin on chondrogenic and hypertrophic differentiation of mesenchymal stem cells. Am. J. Transl. Res. *9*, 2748–2759.

51. Tian, Y., Xu, Y., Xue, T., Chen, L., Shi, B., Shu, B., Xie, C., Max Morandi, M., Jaeblon, T., Marymont, J.V., and Dong, Y. (2017). Notch activation enhances mesenchymal stem cell sheet osteogenic potential by inhibition of cellular senescence. Cell Death Dis. *8*, e2595.

52. Guo, L.Y., Li, Y.M., Qiao, L., Liu, T., Du, Y.Y., Zhang, J.Q., He, W.T., Zhao, Y.X., and He, D.Q. (2012). Notch2 regulates matrix metallopeptidase 9 via PI3K/AKT signaling in human gastric carcinoma cell MKN-45. World J. Gastroenterol. *18*, 7262–7270.

53. Villegas, S.N., Gombos, R., García-López, L., Gutiérrez-Pérez, I., García-Castillo, J., Vallejo, D.M., Da Ros, V.G., Ballesta-Illán, E., Mihály, J., and Dominguez, M. (2018). PI3K/Akt Cooperates with Oncogenic Notch by Inducing Nitric Oxide-Dependent Inflammation. Cell Rep. *22*, 2541–2549.

54. Liu, Z., Ren, Y., Mirando, A.J., Wang, C., Zuscik, M.J., O'Keefe, R.J., and Hilton, M.J. (2016). Notch signaling in postnatal joint chondrocytes, but not subchondral osteoblasts, is required for articular cartilage and joint maintenance. Osteoarthritis Cartilage *24*, 740–751.

55. Lim, H.D., Kim, Y.S., Ko, S.H., Yoon, I.J., Cho, S.G., Chun, Y.H., Choi, B.J., and Kim, E.C. (2012). Cytoprotective and anti-inflammatory effects of melatonin in hydrogen peroxide-stimulated CHON-001 human chondrocyte cell line and rabbit model of osteoarthritis via the SIRT1 pathway. J. Pineal Res. *53*, 225–237.

56. Kita, K., Kimura, T., Nakamura, N., Yoshikawa, H., and Nakano, T. (2008). PI3K/Akt signaling as a key regulatory pathway for chondrocyte terminal differentiation. Genes Cells *13*, 839–850.

57. Rao, Z., Wang, S., and Wang, J. (2017). Peroxiredoxin 4 inhibits IL-1β-induced chondrocyte apoptosis via PI3K/AKT signaling. Biomed. Pharmacother. *90*, 414–420.

58. Ji, Q., Qi, D., Xu, X., Xu, Y., Goodman, S.B., Kang, L., Song, Q., Fan, Z., Maloney, W.J., and Wang, Y. (2018). Cryptotanshinone Protects Cartilage against Developing Osteoarthritis through the miR-106a-5p/GLIS3 Axis. Mol. Ther. Nucleic Acids *11*, 170–179.

59. Evans, C.H., Kraus, V.B., and Setton, L.A. (2014). Progress in intra-articular therapy. Nat. Rev. Rheumatol. *10*, 11–22.

60. Kawanishi, Y., Nakasa, T., Shoji, T., Hamanishi, M., Shimizu, R., Kamei, N., Usman, M.A., and Ochi, M. (2014). Intra-articular injection of synthetic microRNA-210 accelerates avascular meniscal healing in rat medial meniscal injured model. Arthritis Res. Ther. *16*, 488.

61. Peng, J.S., Chen, S.Y., Wu, C.L., Chong, H.E., Ding, Y.C., Shiau, A.L., and Wang, C.R. (2016). Amelioration of experimental autoimmune Arthritis through targeting of synovial fibroblasts by intraarticular delivery of microRNAs 140-3p and 140-5p. Arthritis Rheumatol. *68*, 370–381.

62. Goodrich, L.R., Phillips, J.N., McIlwraith, C.W., Foti, S.B., Grieger, J.C., Gray, S.J., and Samulski, R.J. (2013). Optimization of scAAVIL-1ra in vitro and in vivo to deliver high levels of therapeutic protein for treatment of osteoarthritis. Mol. Ther. Nucleic Acids *2*, e70.

63. Evans, C.H., Ghivizzani, S.C., and Robbins, P.D. (2008). Arthritis gene therapy's first death. Arthritis Res. Ther. *10*, 110.

64. Ding, J., Huang, S., Wu, S., Zhao, Y., Liang, L., Yan, M., Ge, C., Yao, J., Chen, T., Wan, D., et al. (2010). Gain of miR-151 on chromosome 8q24.3 facilitates tumour cell migration and spreading through downregulating RhoGDIA. Nat. Cell Biol. *12*, 390–399.

65. Waki, T., Lee, S.Y., Niikura, T., Iwakura, T., Dogaki, Y., Okumachi, E., Oe, K., Kuroda, R., and Kurosaka, M. (2016). Profiling microRNA expression during fracture healing. BMC Musculoskelet. Disord. *17*, 83.

66. Li, K.C., Chang, Y.H., Yeh, C.L., and Hu, Y.C. (2016). Healing of osteoporotic bone defects by baculovirus-engineered bone marrow-derived MSCs expressing MicroRNA sponges. Biomaterials *74*, 155–166.

67. Genemaras, A.A., Ennis, H., Kaplan, L., and Huang, C.Y. (2016). Inflammatory cytokines induce specific time- and concentration-dependent MicroRNA release by chondrocytes, synoviocytes, and meniscus cells. J. Orthop. Res. *34*, 779–790.

68. Diekman, B.O., Sessions, G.A., Collins, J.A., Knecht, A.K., Strum, S.L., Mitin, N.K., Carlson, C.S., Loeser, R.F., and Sharpless, N.E. (2018). Expression of $p16^{INK4a}$ is a biomarker of chondrocyte aging but does not cause osteoarthritis. Aging Cell *17*, e12771.

69. Santangelo, K.S., Nuovo, G.J., and Bertone, A.L. (2012). In vivo reduction or blockade of interleukin-1β in primary osteoarthritis influences expression of mediators implicated in pathogenesis. Osteoarthritis Cartilage *20*, 1610–1618.

70. Thysen, S., Luyten, F.P., and Lories, R.J. (2015). Targets, models and challenges in osteoarthritis research. Dis. Model. Mech. *8*, 17–30.

71. Liang, Y., Duan, L., Xiong, J., Zhu, W., Liu, Q., Wang, D., Liu, W., Li, Z., and Wang, D. (2016). E2 regulates MMP-13 via targeting miR-140 in IL-1β-induced extracellular matrix degradation in human chondrocytes. Arthritis Res. Ther. *18*, 105.

72. Farr, J.N., Xu, M., Weivoda, M.M., Monroe, D.G., Fraser, D.G., Onken, J.L., Negley, B.A., Sfeir, J.G., Ogrodnik, M.B., Hachfeld, C.M., et al. (2017). Targeting cellular senescence prevents age-related bone loss in mice. Nat. Med. *23*, 1072–1079.

73. Kellgren, J.H., and Lawrence, J.S. (1957). Radiological assessment of osteo-arthrosis. Ann. Rheum. Dis. *16*, 494–502.

74. Meng, F., Zhang, Z., Chen, W., Huang, G., He, A., Hou, C., Long, Y., Yang, Z., Zhang, Z., and Liao, W. (2016). MicroRNA-320 regulates matrix metalloproteinase-13 expression in chondrogenesis and interleukin-1β-induced chondrocyte responses. Osteoarthritis Cartilage *24*, 932–941.

75. Nagase, H., Kumakura, S., and Shimada, K. (2012). Establishment of a novel objective and quantitative method to assess pain-related behavior in monosodium iodoacetate-induced osteoarthritis in rat knee. J. Pharmacol. Toxicol. Methods *65*, 29–36.

76. Kim, S.J., Kim, J.E., Kim, S.H., Kim, S.J., Jeon, S.J., Kim, S.H., and Jung, Y. (2016). Therapeutic effects of neuropeptide substance P coupled with self-assembled peptide nanofibers on the progression of osteoarthritis in a rat model. Biomaterials *74*, 119–130.

77. Jean, Y.H., Wen, Z.H., Chang, Y.C., Lee, H.S., Hsieh, S.P., Wu, C.T., Yeh, C.C., and Wong, C.S. (2006). Hyaluronic acid attenuates osteoarthritis development in the anterior cruciate ligament-transected knee: Association with excitatory amino acid release in the joint dialysate. J. Orthop. Res. *24*, 1052–1061.

78. Wang, C.J., Huang, C.Y., Hsu, S.L., Chen, J.H., and Cheng, J.H. (2014). Extracorporeal shockwave therapy in osteoporotic osteoarthritis of the knee in rats: an experiment in animals. Arthritis Res. Ther. *16*, R139.

79. Huang, M.J., Wang, L., Jin, D.D., Zhang, Z.M., Chen, T.Y., Jia, C.H., Wang, Y., Zhen, X.C., Huang, B., Yan, B., et al. (2014). Enhancement of the synthesis of n-3 PUFAs in fat-1 transgenic mice inhibits mTORC1 signalling and delays surgically induced osteoarthritis in comparison with wild-type mice. Ann. Rheum. Dis. *73*, 1719–1727.

OMTN, Volume *19*

Supplemental Information

miR-140 Attenuates the Progression of Early-Stage Osteoarthritis by Retarding Chondrocyte Senescence

Hai-bo Si, Ti-min Yang, Lan Li, Mei Tian, Li Zhou, Dai-ping Li, Qiang Huang, Peng-de Kang, Jing Yang, Zong-ke Zhou, Jing-qiu Cheng, and Bin Shen

SUPPLEMENTARY DATA

Table S1 General characteristics of the cartilage donors

Groups	N	Age (years)	Gender (male : female)	Side (left : right)	KL grade
Normal	5	48.0 (39, 42, 48, 53, 58)	3: 2	3 : 2	0
E-OA	5	57.8 (51, 52, 60, 61, 65)	2 : 3	3 : 2	1.8 (1, 2, 2, 2, 2)
ML-OA	5	58.6 (51, 58, 59, 62, 63)	2 : 3	2 : 3	3.6 (3, 3, 4, 4, 4)

Abbreviations: E-OA, early-stage OA; ML-OA, middle- to late-stage OA; KL, Kellgren and Lawrence X-ray criteria.

Table S2 The specific primers used for different genes

Genes	Primer	
p16^{INK4a}	Forward	GGCCGATCCAGGTCATGATGATG
	Reverse	CACCAGCGTGTCCAGGAAGC
p21	Forward	GATGGAACTTCGACTTTGTCAC
	Reverse	GTCCACATGGTCTTCCTCTG
p53	Forward	TTCCTGAAAACAACGTTCTGTC
	Reverse	AACCATTGTTCAATATCGTCCG
COL2A1	Forward	GATAACAGTCTTGCCCCACTTA
	Reverse	CAAGAACAGCATTGCCTATCTG
Aggrecan	Forward	GATCCTTACCGTAAAGCCCATC
	Reverse	CTCCAGTCTCATTCTCAACCTC

MMP13	Forward	CACTTTATGCTTCCTGATGACG
	Reverse	TCTGGCGTTTTTGGATGTTTAG
ADAMTS5	Forward	GACCGATGGCACTGAATGTAGGC
	Reverse	TCTCCTCCACATACTCCGCACTTG
β-actin	Forward	5’-GAAGATCAAGATCATTGCTCCT-3’
	Reverse	5’-TGGAAGGTGGACAGTGAG-3’

Table S3 The mature sequences of miR-140-5p in various species

Gene ID	Mature names	Sequence
MIMAT0000431	hsa-mir-150-5p	CAGUGGUUUUACCCUAUGGUAG
MIMAT0000573	rno-mir-150-5p	CAGUGGUUUUACCCUAUGGUAG
MIMAT0000151	mmu-mir-150-5p	CAGUGGUUUUACCCUAUGGUAG
MIMAT0001159	gga-mir-150-5p	AGUGGUUUUACCCUAUGGUAG
MIMAT0001836	dre-mir-150-5p	CAGUGGUUUUACCCUAUGGUAG
MIMAT0002143	ssc-mir-150-5p	AGUGGUUUUACCCUAUGGUAG
MIMAT0006197	mml-mir-150-5p	CAGUGGUUUUACCCUAUGGUAG
MIMAT0006812	oan-mir-150-5p	CAGUGGUUUUACCCUAUGGU
MIMAT0012745	mdo-mir-150-5p	CAGUGGUUUUACCCUAUGGUAG
MIMAT0012926	eca-mir-150-5p	CAGUGGUUUUACCCUAUGGUAG
MIMAT0014557	tgu-mir-150-5p	CAGUGGUUUUACCCUAUGGUAG
MIMAT0015763	ppy-mir-150-5p	CAGUGGUUUUACCCUAUGGUAG
MIMAT0021765	aca-mir-150-5p	CAGUGGUUUUACCCUAUGGU

MIMAT0022552	ola-mir-150-5p	CAGUGGUUUACCCUAUGGUAG
MIMAT0023767	cgr-mir-150-5p	CAGUGGUUUACCCUAUGGUAG
MIMAT0025434	pol-mir-150-5p	CAGUGGUUUACCCUAUGGUAG
MIMAT0026220	ccr-mir-150-5p	CAGUGGUUUACCCUAUGGUAG
MIMAT0032359	ssa-mir-150-5p	CAGUGGUUUACCCUAUGGUAG
MIMAT0035960	chi-mir-150-5p	CAGUGGUUUACCCUAUGGUAG
MIMAT0036560	tch-mir-150-5p	CAGUGGUUUACCCUAUGGUA
MIMAT0036719	oha-mir-150-5p	CAGUGGUUUACCCUAUGGUAG
MIMAT0037764	cpi-mir-150-5p	CAGUGGUUUACCCUAUGGUAG
MIMAT0038160	ami-mir-150-5p	CAGUGGUUUACCCUAUGGUAG
MIMAT0038527	cli-mir-150-5p	CAGUGGUUUACCCUAUGGUAG
MIMAT0039778	pal-mir-150-5p	CAGUGGUUUACCCUAUGGUAG
MIMAT0044299	gmo-mir-150-5p	CAGUGGUUUACCCUAUGGUA
MIMAT0046452	xla-mir-150-5p	CCAGUGGUUUACCCUAUGGUAGG
MIMAT0047004	cpo-mir-150-5p	CAGUGGUUUACCCUAUGGUAG
MIMAT0047653	dno--mir-150-5p	CAGUGGUUUACCCUAUGGUAG
MIMAT0048241	ocu-mir-150-5p	CAGUGGUUUACCCUAUGGUAG

Table S4 The target genes of hsa-miR-140-5p predicted by at least three database*

AARS	CELF2	FBXO45	KLF9	NDST1	PRRG1	SLC41A2	TSC22D2
ABCA1	CEP63	FCHO2	KLK10	NFAT5	PSRC1	SLC44A2	TSPAN12
AC011897.1	CERCAM	FGF9	LAMC1	NFE2L2	PTCD3	SLC6A6	TSSK2

AC026703.1	CMTM6	FNDC5	LAMP2	NFYA	R3HDM1	SMURF1	TTK
ACSL6	CORO2A	FOXP2	LCOR	NLK	RAB10	SNX12	TTYH2
ADAM10	CREB3L1	FXR1	LHFPL2	NPL	RABIF	SNX2	TTYH3
ADAM9	CTCF	GALC	LHX2	NPR3	RALA	SNX27	TULP4
ADAMTS5	CUL3	GALNT16	LIN9	NR1D1	RALGPS1	SOX4	UBE2I
ADCY6	DAG1	GIT1	LMNB1	NRIP1	RALGPS2	SPATA6	UBE2V1
ANK2	DCUN1D3	GLI3	LPHN2	NUCKS1	RBM39	SPOCK1	UBR5
ANKFY1	DDHD2	GNG12	LRAT	NUMBL	REST	SPRED1	VEZF1
ANKIB1	DGKI	GPR161	LRP4	OGT	RFFL	SPRY4	VTI1B
ANKRD12	DNM3	GPR85	LYSMD3	OSBPL6	RFX7	SRCAP	WASF1
ARHGAP19	DOK4	GYS1	MED13	OSTM1	RNF170	ST5	WBP1L
ARL15	DPP10	HAND2	MGAT1	OTX1	RNF19A	STC2	WDFY3
ATG14	DPYSL2	HDAC4	MICAL3	PALM2-AKAP2	ROR1	STK17A	WEE1
BACH1	DTNA	HDAC7	MIER3	PAX9	SATB2	STRADB	WNT1
BAG2	DYNC1LI2	HECTD2	MIPOL1	PDGFRA	SBNO1	SULF1	WNT9A
BAZ2B	EAF1	HEY1	MMD	PFN2	SCARB2	TBC1D12	YES1
BCL11A	EFNA4	HNRNPH3	MOB3A	PGP	SCRN1	TEAD1	YOD1
BCL2L1	EGR2	HRCT1	MSMP	PITX2	SEL1L	TFPI	ZBTB10
BCL2L2	EIF2AK2	HSPA13	MTSS1	PKN2	SEPT2	TGFBR1	ZHX1
BCL9	EIF4G2	HSPA4L	MYCBP2	PLEKHG3	SERPINB1	TIMM23	ZNF608
BIRC6	ELAVL2	IGF1R	MYO10	POLR3G	SH3GL2	TJP1	ZNF800
BMP2	EPB41L2	IGFBP5	MYO6	PPARA	SHROOM3	TLR4	ZNF827

C12orf74	ERBB2IP	IPO7	NAA20	PPP1CC	SIAH1	TMEM123
CALCOCO2	ERC2	JAG1	NAIP	PPP1R12A	SLC25A27	TMEM189-UBE2V1
CALU	FADS1	KAT2B	NAV3	PPP1R9A	SLC2A1	TMEM260
CAND1	FAM175B	KATNBL1	NCKAP1	PPTC7	SLC30A5	TNN
CAPN1	FAM214A	KBTBD2	NCOA1	PRDM1	SLC33A1	TPGS2
CCNYL1	FBN1	KLF6	NCSTN	PRR14L	SLC38A2	TRMT61B

*, The databases used in this study were miRDB, miRmap, PicTar, TargetScan, and DIANA microT-CDS.

Table S5 Kyoto Encyclopedia of Genes and Genome (KEGG) pathway analysis for predicted target genes of hsa-miR-140-5p ($P < 0.05$)

ID	Term	No.	P	Genes
hsa04520	Adherens junction	6	0.003	IGF1R, TJP1, TGFBR1, NLK, WASF1, YES1
hsa05200	Pathways in cancer	14	0.004	IGF1R, WNT1, BMP2, FGF9, TGFBR1, ADCY6, SLC2A1, PDGFRA, RALA, BCL2L1, WNT9A, LAMC1, GNG12, GLI3
hsa05132	Salmonella infection	6	0.007	PFN2, TJP1, DYNC1LI2, WASF1, PKN2, TLR4
hsa04144	Endocytosis	10	0.008	GIT1, IGF1R, DNM3, TGFBR1, PDGFRA, SNX2, SMURF1, SNX12, RAB10, SH3GL2
hsa04151	PI3K-Akt signaling pathway	12	0.010	IGF1R, FGF9, PDGFRA, GYS1, PKN2, CREB3L1, TLR4, TNN, EFNA4, BCL2L1, LAMC1, GNG12
hsa04810	Regulation of actin cytoskeleton	9	0.011	GIT1, PFN2, FGF9, WASF1, PDGFRA, PPP1R12A, GNG12, PPP1CC, NCKAP1
hsa04931	Insulin resistance	6	0.019	PPARA, SLC2A1, GYS1, CREB3L1, OGT, PPP1CC

hsa05166	HTLV-I infection	9	0.029	WNT1, KAT2B, EGR2, TGFBR1, ADCY6, SLC2A1, PDGFRA, BCL2L1, WNT9A
hsa04330	Notch signaling pathway	4	0.031	NCSTN, KAT2B, JAG1, NUMBL
hsa04141	Protein processing in endoplasmic reticulum	7	0.035	BAG2, HSPA4L, YOD1, NFE2L2, EIF2AK2, SEL1L, CAPN1
hsa05217	Basal cell carcinoma	4	0.042	WNT1, BMP2, WNT9A, GLI3
hsa04120	Ubiquitin mediated proteolysis	6	0.047	CUL3, UBR5, BIRC6, SIAH1, UBE2I, SMURF1

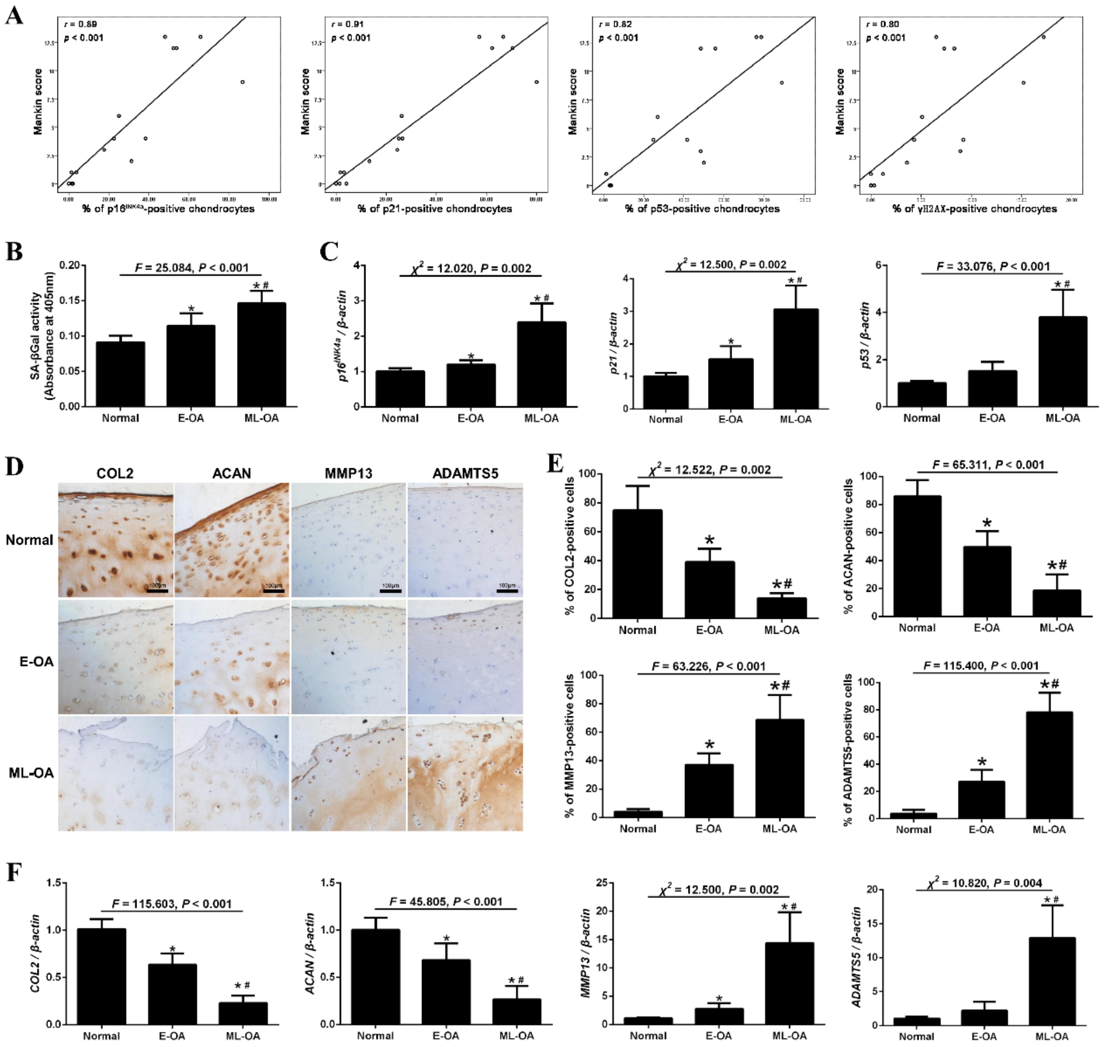

Figure S1. Chondrocyte Senescence and ECM Degradation Correlate with Human OA Pathogenesis

(A) Scatter plots of the positive correlation between the immunopositive chondrocyte percentage of $p16^{INK4a}$, p21, p53 and γH2AX and the modified Mankin score in normal and OA cartilage. (B-C) Basal senescence-associated β-Galactosidase (SA-βGal) activity (B) and gene expression of $p16^{INK4a}$, p21 and p53 (C) in normal and OA cartilage-derived chondrocytes. (D) Representative micrographs of immunohistochemical staining of COL2, ACAN, MMP13 and ADAMTS5 in normal and OA cartilage (scale bar, 100 μm). (E) Percentage of COL2-, ACAN-, MMP13- and

ADAMTS5-positive chondrocytes in normal and OA cartilage. (F) Gene expression of COL2, ACAN, MMP13 and ADAMTS5 in normal and OA cartilage-derived chondrocytes. E-OA, early-stage OA; ML-OA, middle- to late-stage OA. *$p < 0.05$ with respect to normal group, $^{\#}p < 0.05$ with respect to E-OA group.

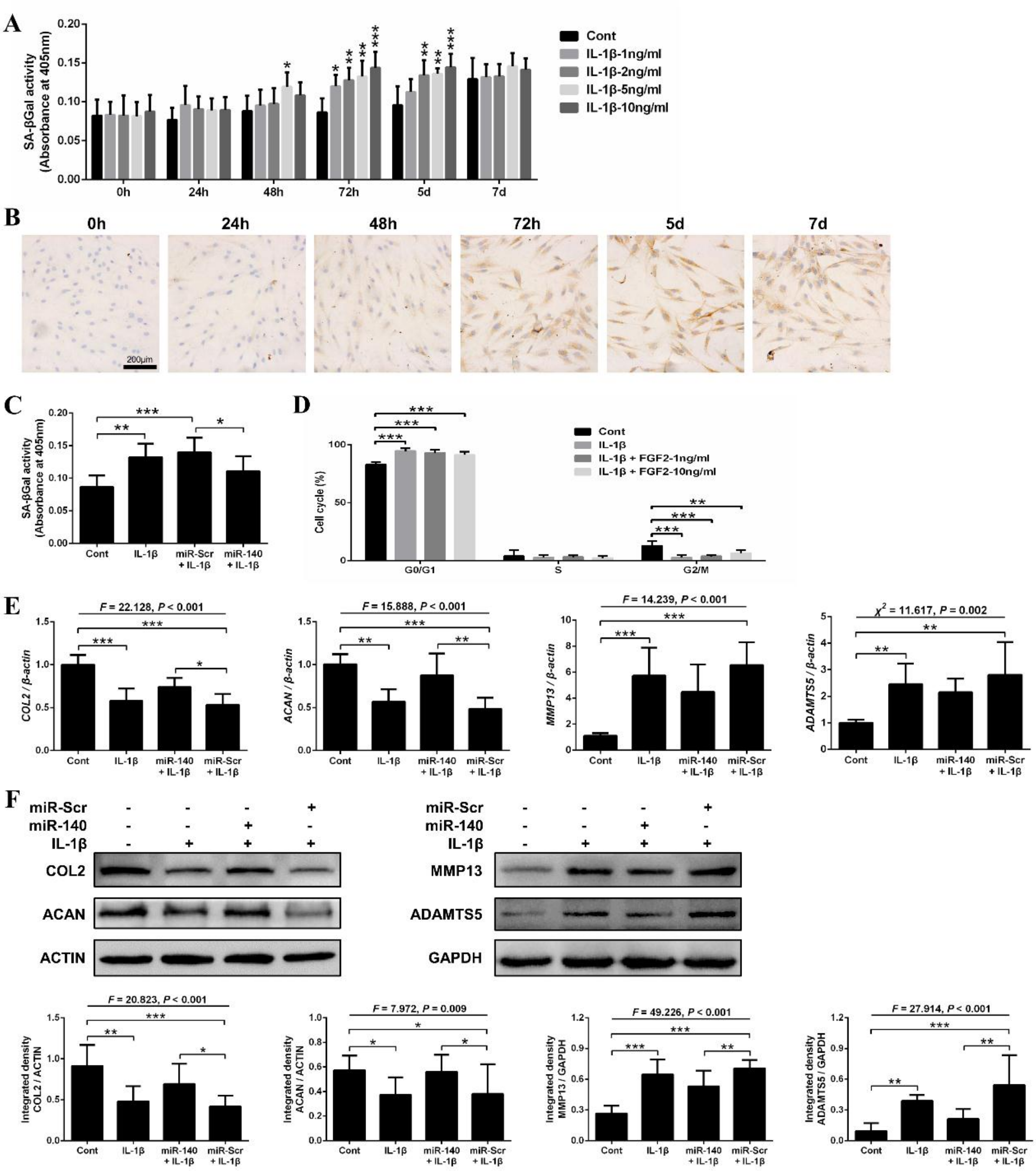

Figure S2. Pretransfection with miR-140 Inhibits IL-1β-induced Human Chondrocyte Senescence and Stabilizes ECM Homeostasis

(A-B) Optimization of IL-1β-induced *in vitro* chondrocyte senescence. *In vitro* chondrocyte senescence was induced by a 24-h IL-1β stimulation in normal chondrocytes, and the results of a time-course change in senescence-associated β-Galactosidase (SA-βGal) activity that included 0 h, 24 h, 48 h, 72 h, 5 d and 7 d after IL-1β treatment with different concentrations (1, 2, 5, 10 ng/ml) showed that 48 h after 5 ng/ml IL-1β stimulation, SA-βGal activity was significantly upregulated (A), while immunocytochemical staining showed that the expression of $p16^{INK4a}$ obviously increased 72 h after 5 ng/ml IL-1β stimulation (B). Thus, 24-h, 5 ng/ml IL-1β treatment was selected for *in vitro* chondrocyte senescence establishment, and a further 48-h incubation in fresh media was allowed before the final analysis. (C) SA-βGal activity in chondrocytes after treatment with normal media (Cont), treatment with IL-1β, pretransfection with miR-Scr + IL-1β, or pretransfection with miR-140 + IL-1β. (D) Percentage of chondrocytes in the G0/G1, S and G2/M phases of the cell cycle after treatment with normal media (Cont), IL-1β, IL-1β + FGF2-1 ng/ml, or IL-1β + FGF2-10 ng/ml. (E-F) Gene and protein expression of COL2, ACAN, MMP13 and ADAMTS5 in chondrocytes after treatment with normal media (Cont), treatment with IL-1β, pretransfection with miR-Scr + IL-1β, or pretransfection with miR-140 + IL-1β. *$p < 0.05$, **$p < 0.01$, ***$p < 0.001$.

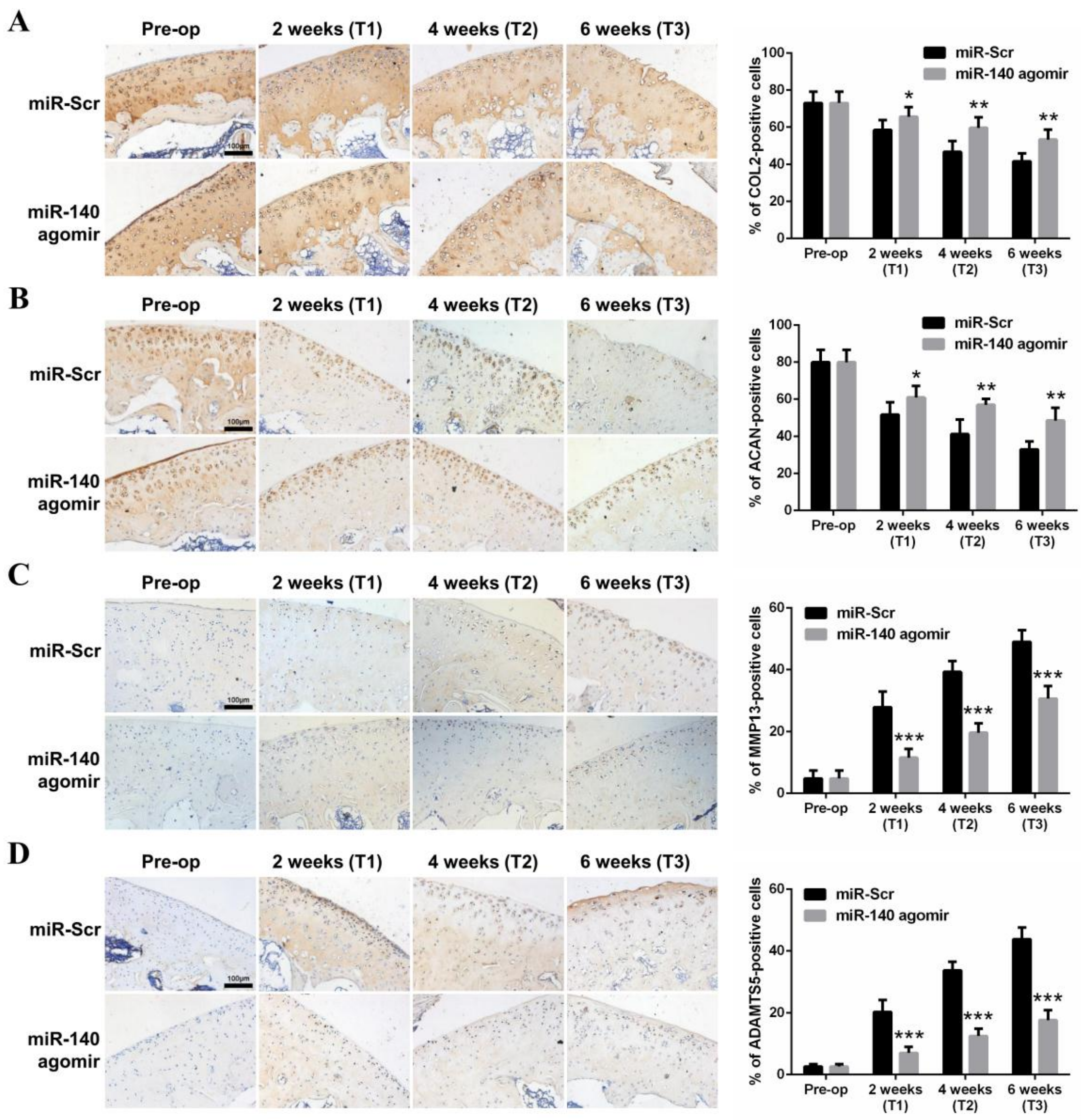

Figure S3. Intra-articular Injection (IAJ) of miR-140 Stabilizes ECM Homeostasis in Rats

(A-D) Representative micrographs of immunohistochemical staining and the immunopositive percentage of COL2 (A), ACAN (B), MMP13 (C) and ADAMTS5 (D) in sagittal sections of the medial femoral condyles after IAJ of miR-Scr or miR-140 agomir in rats (scale bar, 100 μm). * $p < 0.05$, ** $p < 0.01$, *** $p < 0.001$.

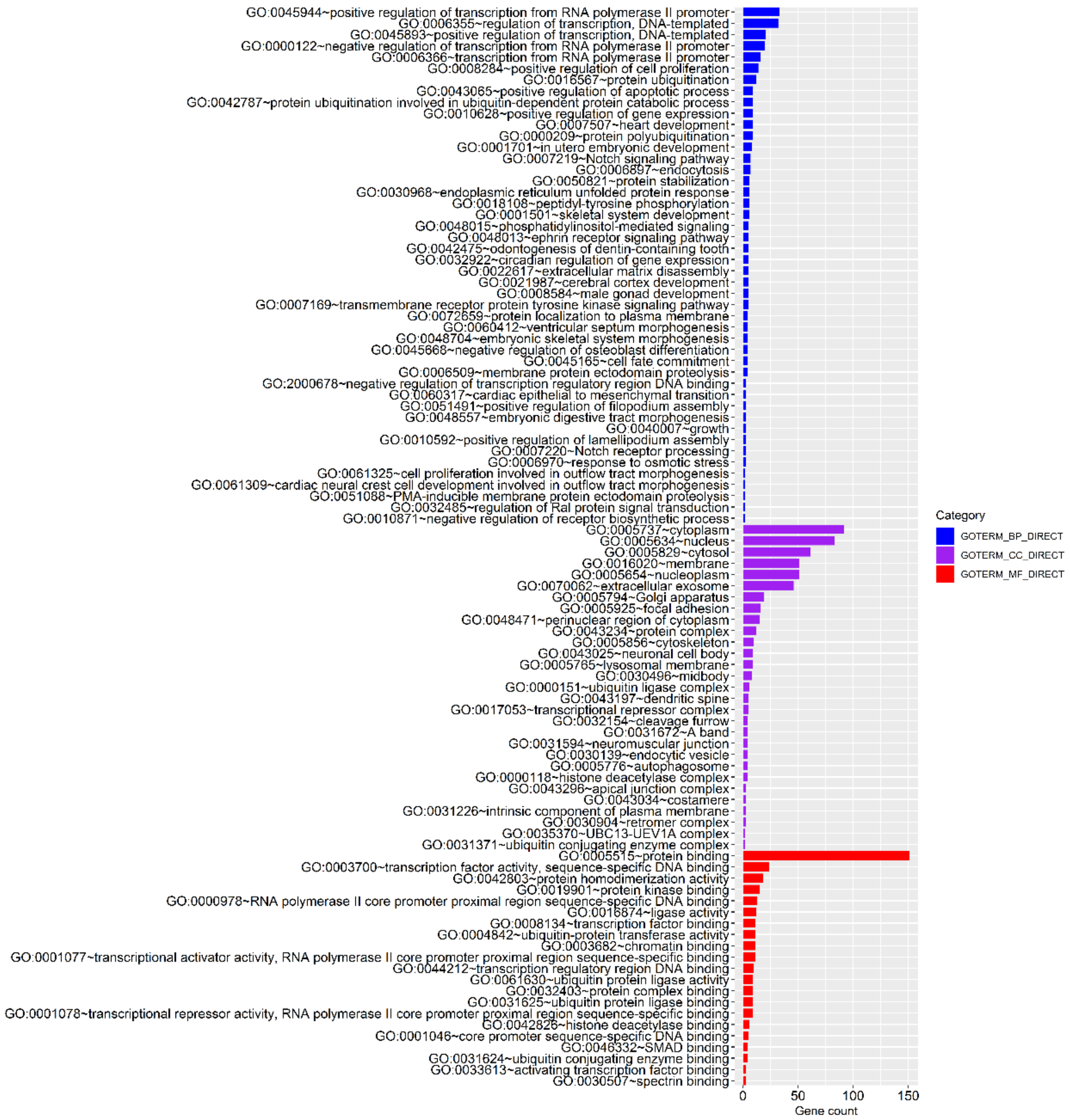

Figure S4. Gene Ontology (GO) Enrichment Analysis for the Predicted Target Genes of hsa-miR-140-5p (All Items in Each Category are Shown)

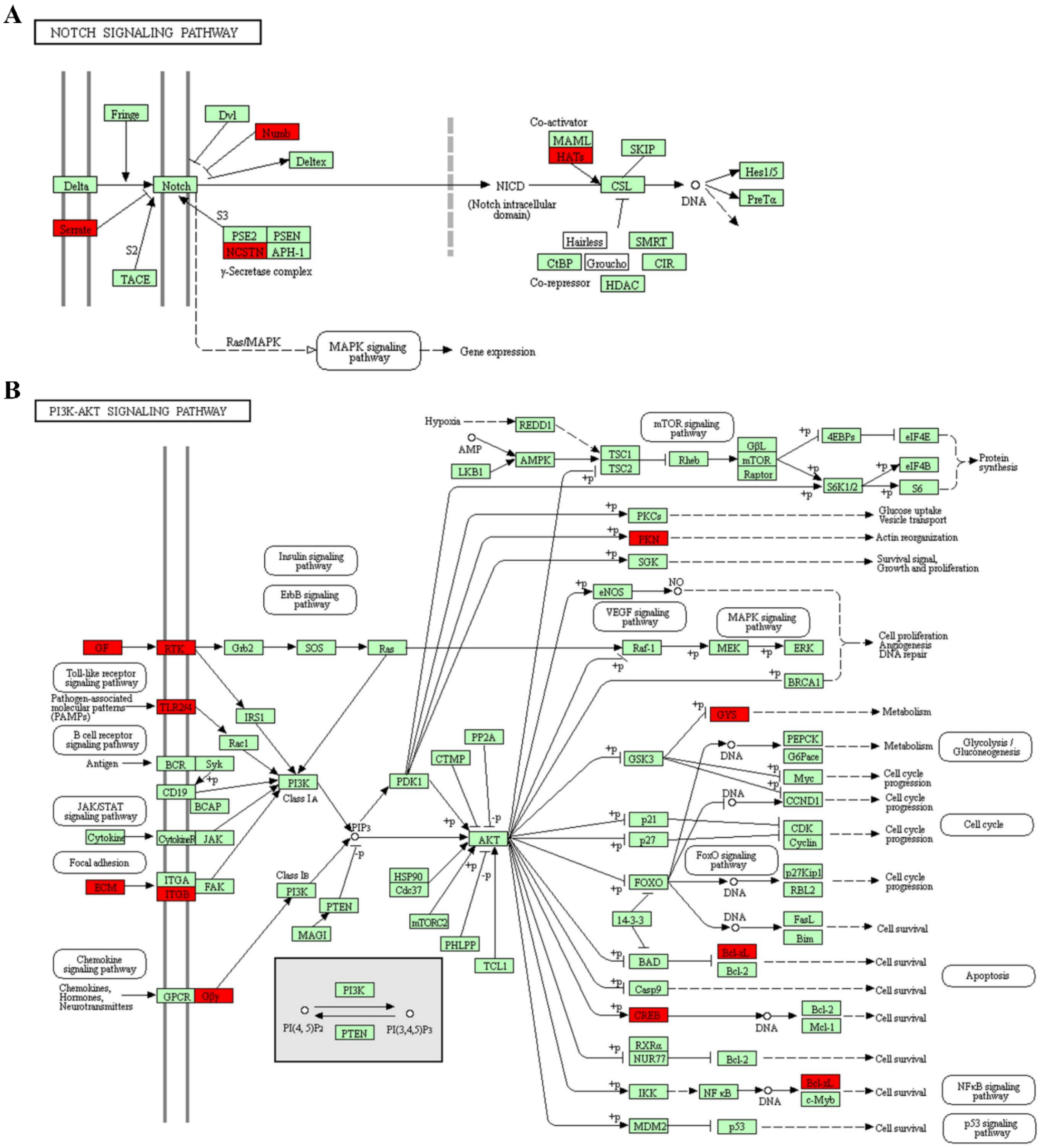

Figure S5. Signaling Pathway Information Generated by Kyoto Encyclopedia of Genes and Genomes (KEGG) Analysis

(A) Notch signaling pathway network generated by KEGG analysis. JAG1, NCSTN, KAT2B and NUMBL were predicted as target genes of hsa-miR-140-5p and enriched in the Notch pathway (located in the red region). (B) PI3K-AKT signaling pathway network generated by KEGG analysis. IGF1R, FGF9, PDGFRA, GYS1, PKN2, CREB3L1, TLR4, TNN, EFNA4, BCL2L1, LAMC1 and GNG12 were predicted as

target genes of hsa-miR-140-5p and enriched in the PI3K-AKT pathway (located in the red region).

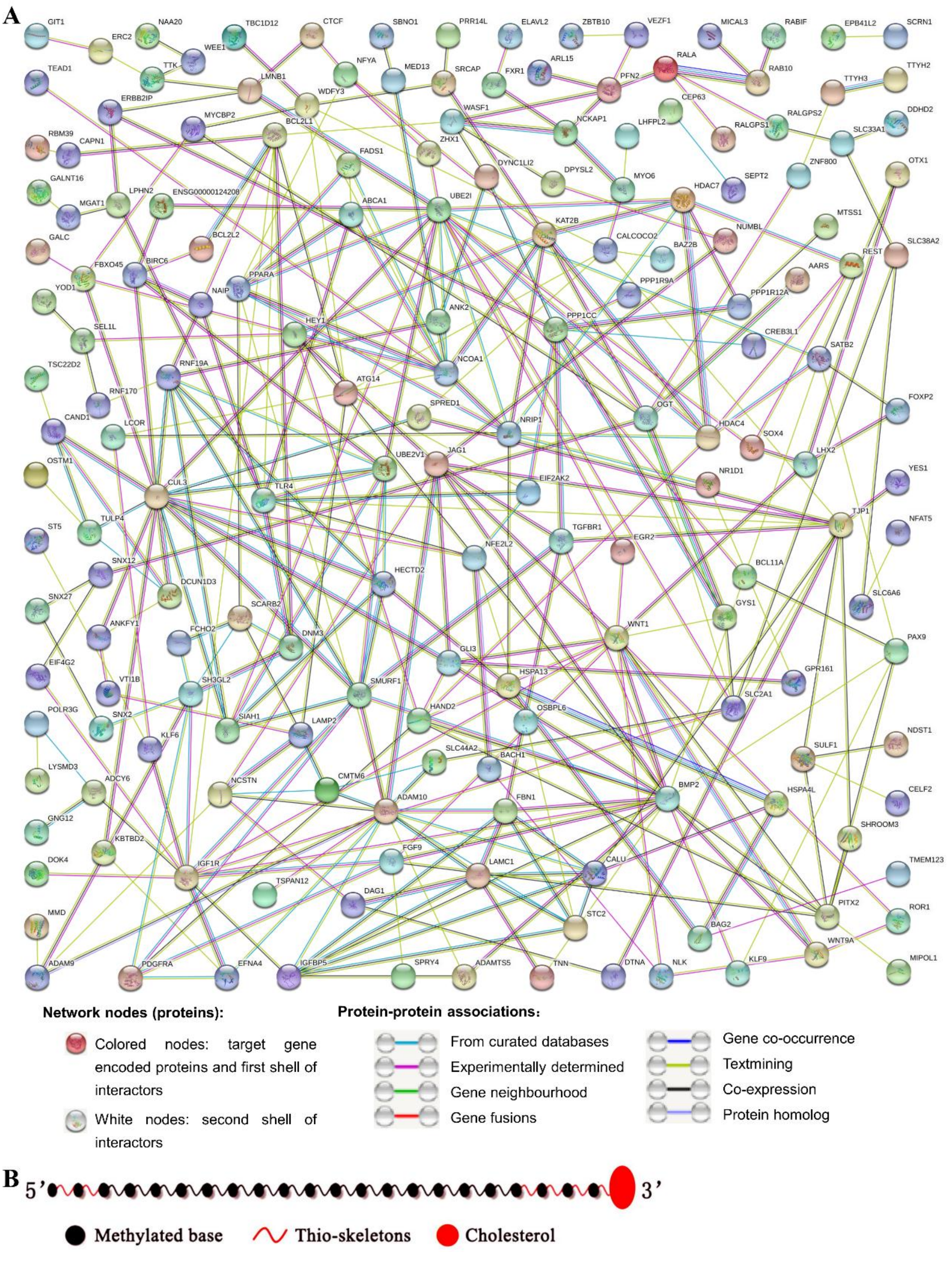

Figure S6. The Interactive Relationships among the Proteins Encoded by the

Target Genes of hsa-miR-140-5p and a Diagram of the Specifically Modified miR-140 Agomir

（A）The interactive relationships among the proteins encoded by the target genes of hsa-miR-140-5p. (B) Diagram of the specially modified hsa-miR-140 agomir. The antisense chain of the synthetic double-stranded miR-140 was fully modified by the addition of methoxyl group, the 3’ end was modified by the addition of cholesterol and thio-skeletons, and the 5’ end was modified by the addition of thio-skeletons.
